# Supplementary material for: Mapping the global research landscape of mitophagy in Parkinson's disease: a bibliometric and visualization analysis
Source: Hereditas. 2025 Nov 20;162:230. doi: 10.1186/s41065-025-00544-y (PMC12632019; doi:10.1186/s41065-025-00544-y)
Supplement: Supplementary file 1 — Additional file 1: Mapping the Global Research Landscape of Mitophagy [file 41065_2025_544_MOESM1_ESM.docx]

Mapping the global research landscape of mitophagy in Parkinson's disease: a bibliometric and visualization analysis

Junqiao Zhao^1^, Qian Wang^1^, Yan Cao^1^, Huimin Shan^2^, Shifen Xu^1*^

^1^Shanghai Municipal Hospital of Traditional Chinese Medicine, Shanghai University of Traditional Chinese Medicine, Shanghai, China. ^2^Harvard Medical School, Brigham and Women’s Hospital, Boston, USA. [*xu_teacher2006@126.com](mailto:*xu_teacher2006@126.com)

Clinical trial number: not applicable.

**Abstract**

Parkinson’s disease (PD) is closely linked to mitochondrial dysfunction and mitophagy, a key mechanism in PD pathogenesis. However, no dedicated bibliometric analysis of mitophagy in PD exists. This study used data from the Web of Science Core Collection to map the global research landscape of mitophagy in PD. The analysis of 1,578 publications (2007–2024) identifies the United States as the most productive country. McGill University ranks as the top institution, and Nobutaka Hattori is the most prolific author. The journal *Autophagy* is the journal with the highest number of publications in this field. Core research themes included PINK1/Parkin, mitochondrial quality control, α-synuclein, neuroinflammation, and ferroptosis. The study provides insights into the current status of global collaboration and translational progress in this field. Future efforts should aim to further explore new pathways, enhance clinical translation, and promote collaborative partnerships to advance research and address challenges in the field.

Keywords: Parkinson’s disease; Mitophagy; Bibliometric analysis; PINK1/Parkin

# **Introduction**

Parkinson's disease (PD), recognized as the fastest-growing neurodegenerative disorder globally, currently affects over 11.7 million individuals worldwide[1]. Characterized by progressive motor dysfunction and debilitating non-motor symptoms, PD imposes substantial socioeconomic burdens, evidenced by the $51.9 billion total economic burden recorded in the U.S. in 2017[2]. Global projections indicate a concerning 1.5-fold increase in PD prevalence by 2035, primarily driven by demographic aging trends[1]. While epidemiological studies highlight geographical variations in disease burden, China's projected contribution to the global PD population warrants particular attention, with estimates suggesting it may account for nearly half of worldwide cases by 2030[3]. This escalating public health challenge underscores the urgent need for multinational collaborative research and innovative therapeutic strategies. Neuropathologically, PD is characterized by the progressive loss of dopaminergic neurons in the substantia nigra pars compacta (SNpc) and the accumulation of misfolded α-synuclein in Lewy bodies and lewy neurites[4,5]. Although the etiology of PD has not yet been fully elucidated, accumulating evidence indicates that mitochondrial dysfunction plays a central role in disease progression. Mitochondria are essential for neuronal survival due to their vital role in energy production[6]. In dopaminergic neurons, mitochondrial dysfunction impairs ATP production, contributing to PD pathogenesis by triggering neurodegeneration and compromising calcium buffering, redox balance, and apoptosis[7].Notably, the pioneering discovery by Schapira's team in 1989 first reported significantly reduced activity of mitochondrial respiratory chain Complex I in postmortem brain tissues of PD patients, establishing an important molecular link between mitochondrial impairment and PD pathogenesis[8]. This discovery established the fundamental basis for the "mitochondrial deficiency hypothesis".

Subsequent genetic investigations have further corroborated this framework by demonstrating direct associations between pathogenic mutations in mitochondrial quality control genes (notably PINK1 and Parkin) and familial PD cases[9,10]. PINK1/Parkin-linked PD typically presents with early-onset, slower progression, and minimal cognitive decline compared to idiopathic PD, which predominantly affects older individuals with accelerated cognitive deterioration[11][12][13]. Notably, while murine models lacking PINK1 or Parkin exhibit only subtle phenotypes, Drosophila genetics played a pivotal role in establishing mitophagy, as loss-of-function mutations in these genes caused severe mitochondrial dysfunction and dopaminergic neurodegeneration mimicking human PD[14]. PD animal models induced by mitochondrial toxins (e.g., MPTP and rotenone) provide valuable insights into acute toxicity leading to dopaminergic neuron degeneration in the substantia nigra[15]. However, there are key differences compared to human PD, particularly in neuropathology and the relative absence of protein inclusion pathology such as Lewy bodies[16]. Despite these differences, these models still highlight the importance of mitochondrial dysfunction in PD pathogenesis. The accumulation of damaged mitochondria can trigger neuronal death, highlighting the importance of efficient mitochondrial quality control mechanisms[17].

Mitophagy, the targeted degradation of mitochondria via autophagy, is a key process for maintaining cellular health. The pathogenesis of PD is closely associated with dysfunctional mitophagy. This process is precisely regulated through both PINK1/Parkin-dependent and PINK1/Parkin-independent pathways. When mitochondrial membrane potential (Δψm) is lost, the serine/threonine kinase PINK1(PTEN-induced putative kinase 1) is no longer imported and cleaved. Instead, it accumulates on the outer mitochondrial membrane (OMM). There, it recruits and phosphorylates the E3 ubiquitin ligase Parkin[19]. Activated Parkin then ubiquitinates numerous OMM proteins (e.g., mitofusins, TOM20, VDAC1)[20], creating phosphorylated ubiquitin chains. These chains are decoded by autophagic receptors such as NDP52, OPTN, and NBR1. These receptors simultaneously engage LC3/GABARAP on the forming phagophore and recruit the core autophagy machinery to drive selective engulfment and lysosomal degradation of the damaged organelle[21][22]. In parallel, PINK1/Parkin-independent mitophagy is executed by a diverse set of receptors—BNIP3, NIX/BNIP3L, and FUNDC1—that are constitutively present or stress-inducibly expressed on the OMM and directly bind LC3 via LC3-interacting regions[23]. Furthermore, lipid-mediated pathways, in which cardiolipin externalization or ceramide clustering on the OMM serves as an “eat-me” signal, also contribute to mitophagy[23][24]. Additionally, mitochondrial-derived vesicles (MDVs) can bud off and deliver oxidized cargo to lysosomes for microautophagy[25]. Together, these interlocking surveillance systems ensure rapid recognition, segregation, and disposal of dysfunctional mitochondria. Their convergence or failure ultimately determines neuronal survival in PD.

In addition to genetic determinants, environmental factors and aging critically impair mitophagic function. With advancing age, the progressive decline in mitochondrial function and reduced autophagic capacity lead to intracellular accumulation of damaged mitochondria, which may constitute a key pathogenic mechanism in PD[26]. In PD therapeutic research, intervention strategies targeting mitophagy have gradually become an important research direction. Several recently developed small-molecule compounds, such as those activating PINK1 or Parkin, have demonstrated potential for enhancing mitophagy in preclinical models, offering new therapeutic prospects for PD[27]. In conclusion, mitophagy plays a central role in the pathogenesis of PD. Elucidating the regulatory mechanisms of mitophagy and its dysfunction in neurodegenerative contexts not only deepens our understanding of PD pathology, but also establishes a critical theoretical framework for developing novel therapeutic interventions targeting mitochondrial quality control.

The growing scientific interest in mitophagy's role in PD pathogenesis underscores the critical need for systematic literature analysis. Comprehensive evaluation of research evolution is essential for understanding how research trends have evolved, identifying gaps in knowledge, and guiding future research efforts. In contrast to traditional reviews, bibliometric analysis provides distinct methodological advantages: it enables efficient processing of large-scale literature datasets, quantitative identification of research hotspots through citation network analysis, and visual mapping of conceptual relationships, thereby eliminating the need for labor-intensive manual article evaluations. This study employs integrated bibliometric and scientometric visualization techniques to systematically examine global research patterns in PD-related mitophagy through 2024. By analyzing publication metrics, citation networks, and collaborative patterns, our approach traces the historical development of mitophagy research while identifying emerging therapeutic paradigms. It serves as a valuable roadmap for researchers, clinicians, and funding agencies, while also emphasizing the significant achievements and collaborative efforts that have influenced this field of study.

# **Materials and methods**

## **Data collection**

This study primarily utilized the Web of Science Core Collection (WoSCC; Clarivate Analytics, USA) - the gold-standard multidisciplinary citation index encompassing over 34,000 peer-reviewed journals across 254 research categories since 1900 - to ensure rigorous bibliometric analysis of high-impact scientific literature. (Platform access: https://www.webofscience.com/wos). To systematically map the global research landscape of mitophagy in PD, the following search formula was applied: TS=(Parkinson* OR PD) AND TS=(mitophagy OR "mitochondrial autophagy"). The search was limited to articles and reviews published up to December 2024. The final search was conducted on January 12, 2025, to ensure inclusion of the most recent publications and to prevent data bias due to database updates. A total of 1712 documents were retrieved.

The initial search yield was filtered to include only English-language publications. Editorial material, book chapters, early access, meeting abstract, proceeding paper, correction, letter, data paper, publication with expression of concern, and retracted publication were excluded. The final dataset comprised articles and reviews articles that specifically addressed aspects of mitophagy in the context of PD.

## **Bibliometric analysis**

To strengthen the validity and multidimensional scope of our investigation, we employed several bibliometric tools to uncover trends and patterns within this field.

CiteSpace, a software developed by Chaomei Chen, was pivotal in visualizing the co-citation and co-authorship networks, as well as tracking the evolution of key terms within the field[28]. Our utilization of CiteSpace 6.4 R1 allowed us to delve into hotspots countries/regions, dual-map overlays of journals, keyword timelines, and co-citation analyses.

In conjunction with CiteSpace, VOSviewer, developed by Nees Jan van Eck and his team, was instrumental in the bibliometric network graph analysis[29]. In this study, we utilized VOSviewer (version 1.6.20; Centre for Science and Technology Studies, Leiden University, The Netherlands) to visualize the distribution of countries/regions, institutions, and journals, and to construct co-authorship and keyword co-occurrence networks. The clustering algorithm of VOSviewer, which is based on a similarity matrix and the VOS mapping technique, enabled the automated clustering process. We also employed Pajek (version 5.18; developed by Andrej Mrvar and Vladimir Batagelj, Faculty of Computer and Information Science, University of Ljubljana) in conjunction with VOSviewer. Pajek is an advanced tool for network analysis, specializing in the visualization and management of large-scale networks[30]. In our research, Pajek was used to calculate and optimize graph data and correlation networks, thereby enhancing the clarity and interpretability of the network visualizations.

To further enhance our analysis, we employed Bibliometrix, an R-based tool, to examine key bibliometric data[31]. For the visualization and predictive modeling of publication trends over time, including both annual and predicted publication volumes, we utilized OriginPro 2024 software (OriginLab Corporation, 2024).

## **Ethical Considerations**

This study is a bibliometric analysis, and all data were obtained from publicly available academic database. As the research did not involve human or animal experimentation, personal data collection, or sensitive information, ethical approval was not required. All data were aggregated and analyzed in compliance with database terms of use, copyright regulations, and academic integrity standards to ensure the transparency and reproducibility of the study.

## **Results**

## **Literature overview**

Following the screening protocol and publication search process depicted in Figure 1, we identified 1,578 publications related to mitophagy in PD, including 1,082 articles and 496 reviews. Using Bibliometrix, we conducted a detailed bibliometric analysis, revealing a 34.79% annual growth rate and a robust body of literature. A total of 7,428 authors contributed to these publications in 453 sources. There are only 33 single-authored documents, and the average number of co-authors per document is 6.9. Furthermore, the international co-authorship rate is 26.43%. The average age of a document is 6.25 years, and the average citation count per document is 72.85.

**Fig.1**

Literature screening process and results for bibliometric analysis of mitophagy research in PD


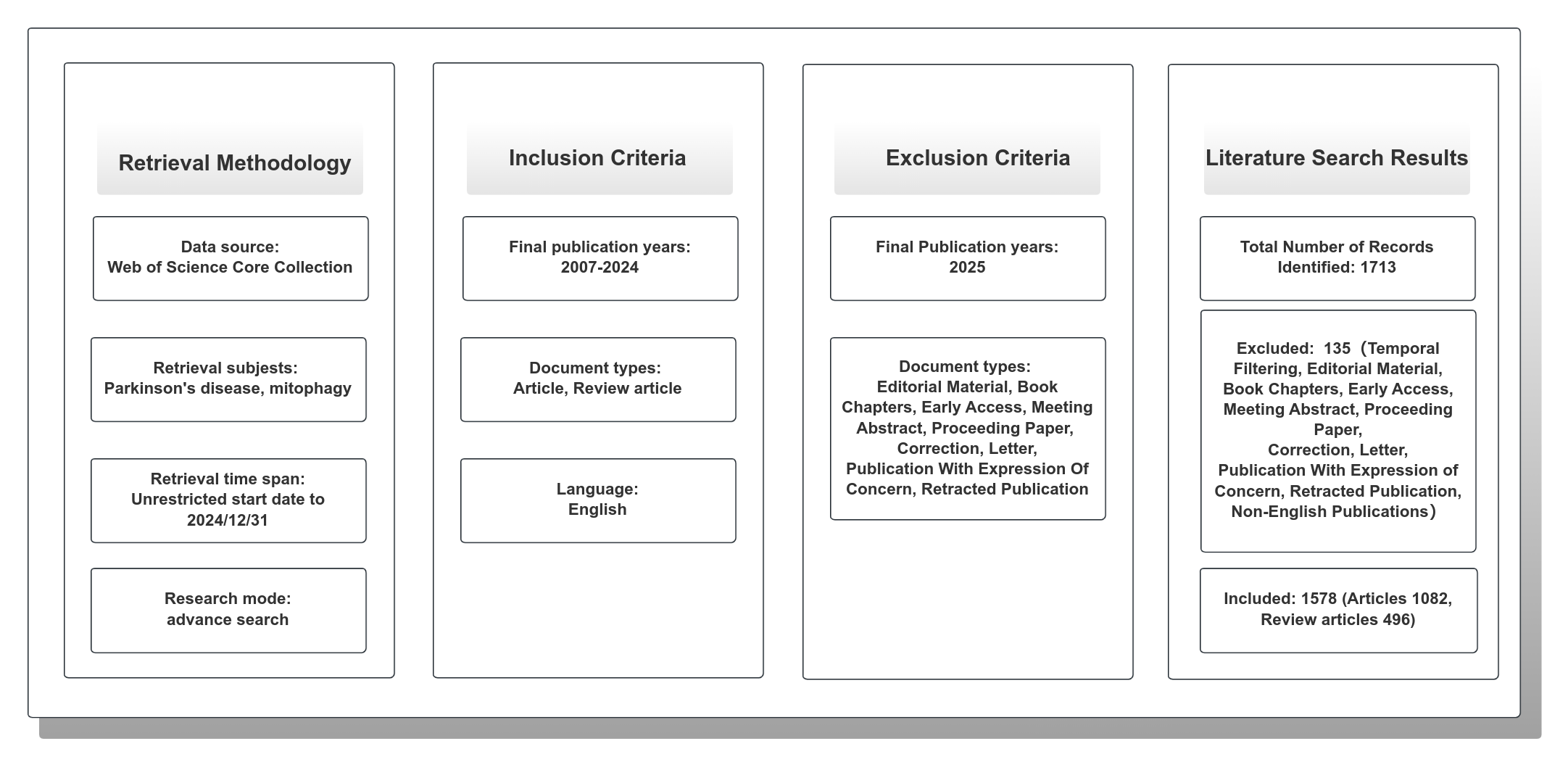


## **Publication outputs and trends**

Figure 2A shows the annual and cumulative publication trends from 2007 to 2024. The first publication appeared in 2007, and since then, the number of publications has increased significantly, reaching a peak of 178 publications in 2021. The cumulative publications, shown by the dark blue line, demonstrate a steady upward trend, reflecting the growing body of research in this area.

Figure 2B presents a nonlinear fitting curve generated using OriginPro 2024 software, which models the logistic growth of annual publications in this field from 2007 to 2024. The curve, derived from logistic regression analysis, is displayed alongside the actual publication data points and exhibits a strong correlation with an R-Square value of 0.9422, indicating a reliable fit. It forecasts a sustained rise in the number of publications, estimating around 225 publications per year by 2040. This projection is depicted by a red curve, accompanied by a 95% prediction band and a 95% confidence band, which visually represent the uncertainty associated with the predictions by accounting for data variability.

**Fig. 2**

Description and forecast of publication volume for mitophagy-related research in PD. **A** The chart illustrates the annual publication volume (purple bar chart) and cumulative publication volume (dark blue line chart) related to mitophagy in PD from 2007 to 2024, showing an overall increasing trend, with the highest number of publications in 2021. The number of publications ranges from a minimum of 1 in 2007 to a maximum of 178 in 2021, with an average of 87.67 and a standard deviation of 58.27. **B** The left chart depicts the forecasted annual number of publications from 2025 to 2040. It presents the logistic fit model, along with the 95% prediction band and the 95% confidence band. The parameters of the logistic model are A1 = -74.62532, A2 = 232.91223, x0 = 2014.64653, and p = 302.71514, with an R-Square value of 0.9422, indicating a high degree of model fit. This model suggests a continuous increase in the volume of publications, projecting approximately 225 publications annually by 2040. The right chart is a zoomed-in view within the dashed line frame for clarity


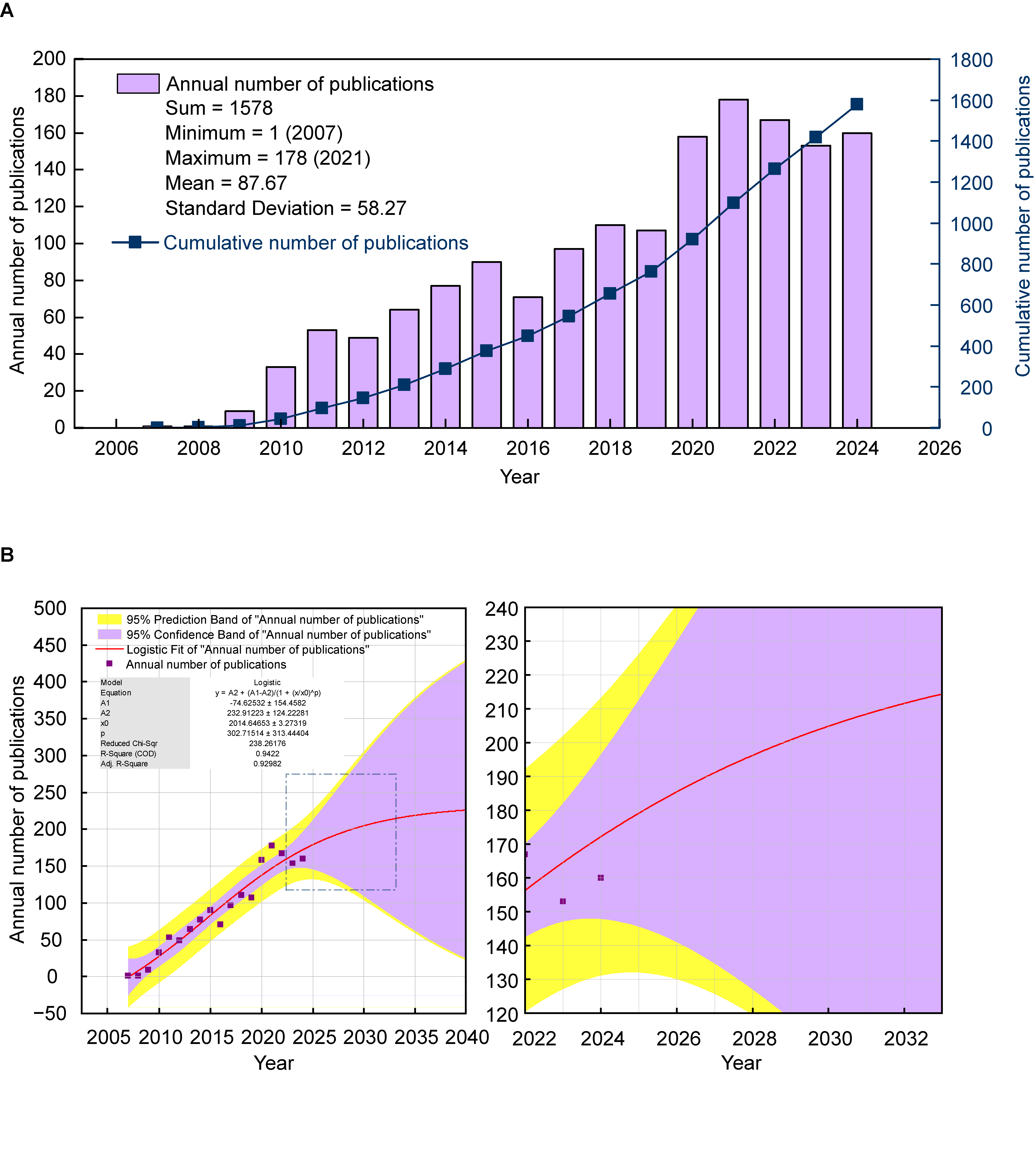


## **Analysis of countries/regions and cooperation relationships**

Currently, a total of 69 countries/regions are actively engaged in research on mitophagy in PD, with a primary focus in the Northern Hemisphere, particularly in North America, Europe, and East Asia. Table 1 lists the top 10 countries/regions in terms of publication volume. The betweenness centrality measures the significance of these countries/regions as intermediaries, highlighting their connectivity and influence. The Total link strength indicates the cumulative strength of all connections that a country or region has with others, reflecting both the quantity and intensity of collaborations. A higher Total link strength suggests more frequent and impactful partnerships.

**Table 1**

Top 10 Countries/regions by publication volume including centrality and total link strength

| Rank | Countries/regions | Count (%) | Centrality | Total link strength |
| --- | --- | --- | --- | --- |
| 1 | USA | 479 (30.35%) | 0.58 | 312 |
| 2 | China | 353 (22.37%) | 0.13 | 102 |
| 3 | England | 170 (10.77%) | 0.24 | 188 |
| 4 | Germany | 122 (7.73%) | 0.08 | 153 |
| 5 | Italy | 121 (7.67%) | 0.09 | 99 |
| 6 | Canada | 95 (6.02%) | 0.12 | 90 |
| 7 | Japan | 89 (5.64%) | 0.03 | 31 |
| 8 | India | 63 (3.99%) | 0.05 | 26 |
| 9 | South Korea | 62 (3.93%) | 0.04 | 19 |
| 10 | Spain | 49 (3.11%) | 0.08 | 61 |

The USA leads with the most publications (479, 30.35%) and a betweenness centrality of 0.58—more than double that of the second, England (0.24), and far above others. This highlights the USA's key role in the global research network and its significant impact on mitophagy research in PD. China, with 353 (22.37%) publications and an intermediary centrality of 0.13, also figures prominently, reflecting its growing impact on global scientific discourse. England (170, 10.77%) and Germany (122, 7.73%) further underscore the significant European contribution to this research domain. However, the lower intermediary centrality values for these countries/regions suggest that their role in bridging international collaborations could be further enhanced.

Figure 3A and B displays the cooperation map and chord diagram of countries/regions. The USA has the highest number of collaborative partners, largely cooperating with China, England, Germany, Canada, Italy, Japan, South Korea, India, France, Australia, Denmark, Sweden, and Poland. China closely cooperates with England, Singapore, Japan, Germany, Italy, Canada, India, Sweden, and France. England frequently cooperates with Germany, Italy, Canada, Spain, France, Portugal, the Netherlands, and Australia.

Figure 3C provides an overview of the top 9 countries/regions with the strongest citation bursts, sorted by the start time of their respective bursts. A citation burst signifies a period during which a country or a region’s scientific publications receive a sudden and significant increase in citations. The USA began its significant burst in 2007, while China and India both demonstrate notable bursts from 2022 to 2024, with China's burst strength reaching 20.96. These bursts reflect critical periods of intense research activity and influence within the field.

Figure 3D presents the annual publication trends for the USA, China, and India, which are among the countries/regions with the highest publication volumes and significant citation bursts. The USA shows a consistent increase in publication numbers over the years, with fluctuations but an overall upward trend, peaking around 2022. China's trend shows a steady rise from 2018 onwards, aligning with the table data that indicates a period of rapid growth in research output and influence. India, starting with a lower base,shows a gradual increase in publications, with a more pronounced rise from 2020.

**Fig.3**

**Country/region analysis of mitophagy in PD. A** Visualization of the collaborative network among all 69 countries/regions using VOSviewer. Node size shows research involvement, color denotes clusters, and line thickness indicates collaboration strength. **B** Chord diagram of international collaborations, with arc width indicating collaboration strength. **C** Top 9 countries/regions with citation bursts, showing years and burst strengths. **D** Publication trends for USA, China, and India from 2007 to 2024, with lines representing countries and axes showing years and publication numbers.


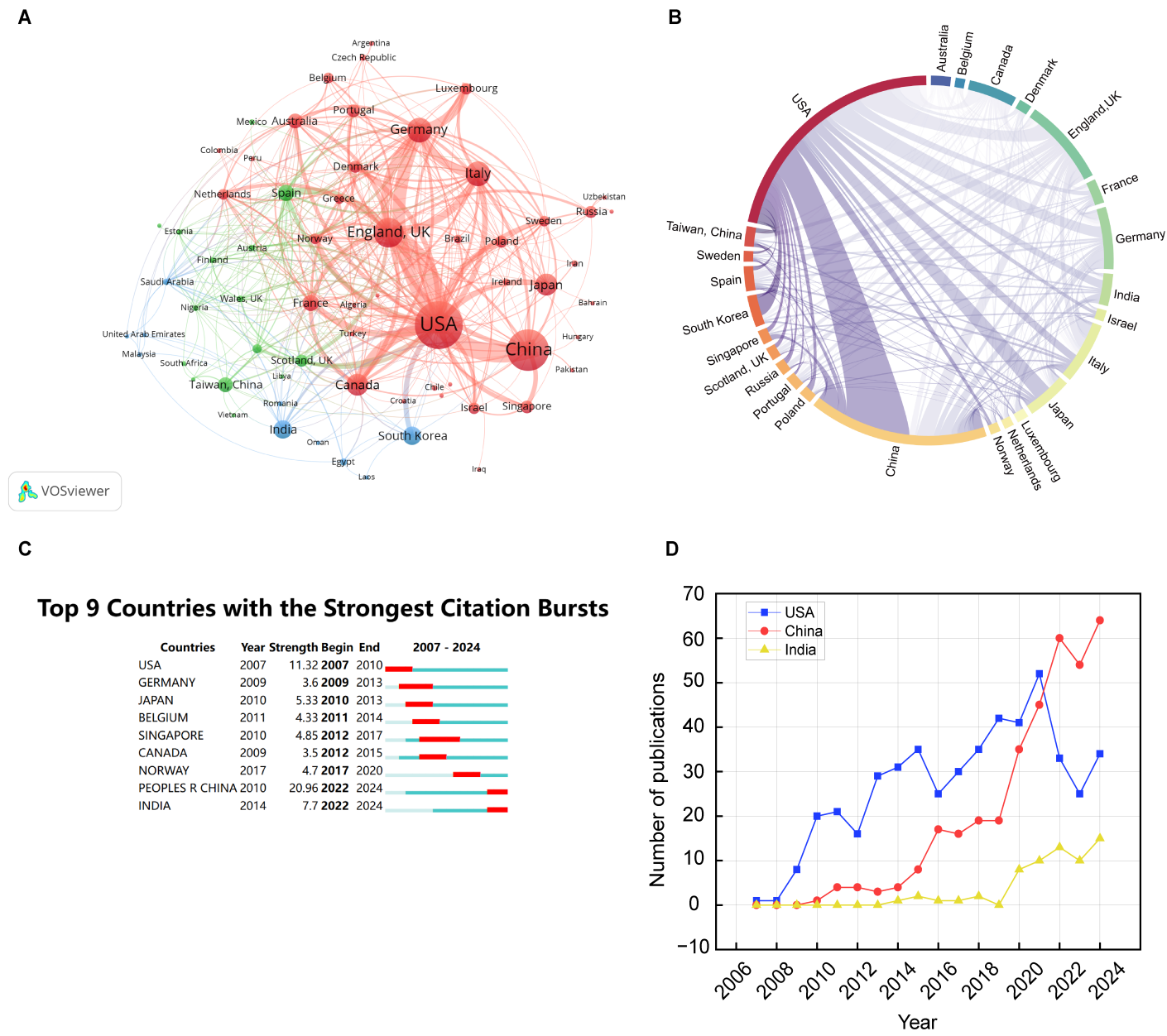


## **Analysis of and institutional cooperations**

Table 2 ranks the top 10 institutions by publication count. McGill University leads with 52 publications, followed by University College London and the University of Pittsburgh with 45 and 38 publications, respectively. Among these top-ranked institutions, four are from the United States, the most represented country, followed by Germany which has two institutions represented. In addition to publication counts, total link strength (which quantifies collaborative connections within the research network) indicates that University College London has the highest level of collaborative engagement, with a score of 128.

**Table 2**

Top 10 institutions by publication volume including citations and total link strength

| Rank | Institution | Country/Region | Publication | Citations | Total link strength |
| --- | --- | --- | --- | --- | --- |
| 1 | McGill University | Canada | 52 | 5329 | 84 |
| 2 | University College London | England | 45 | 3791 | 128 |
| 3 | University of Pittsburgh | USA | 38 | 3953 | 44 |
| 4 | Juntendo University | Japan | 34 | 4027 | 55 |
| 5 | Mayo Clinic | USA | 30 | 1752 | 99 |
| 6 | Chinese Academy of Sciences | China | 29 | 1268 | 77 |
| 7 | National Institute of Neurological Disorders and Stroke | USA | 27 | 10427 | 61 |
| 8 | **University of Lübeck** | **Germany** | 26 | 1899 | 72 |
| 9 | Johns Hopkins University | USA | 25 | 4827 | 83 |
| 10 | University of Tübingen | Germany | 23 | 3506 | 119 |

Figure 4A presents an institutional collaboration network map generated using VOSviewer. This network comprises 180 institutions, each depicted as a node, selected based on having published at least five papers, while excluding those lacking partnerships with other institutions. The resultant chart delineates five distinct clusters, with each node's color corresponding to its specific group. Node sizes are proportional to the number of publications, which highlights institutions with higher research output. The thickness of the lines connecting the nodes reflects the extent of collaboration, with thicker lines signifying more frequent cooperative interactions. Upon analyzing the network, it is evident that certain institutions occupy central positions within the diagram, such as McGill University, University College London, University of Pittsburgh, Mayo Clinic, and the National Institute of Neurological Disorders and Stroke. This indicates their crucial role in the overall collaborative network. These central nodes, distinguished by their larger size and numerous connections, likely represent key research hubs actively involved in knowledge exchange and joint research initiatives. Furthermore, it is apparent that institutions with close collaborative relationships predominantly belong to the same country/region.

Figure 4B presents a network map with nodes identical in size and spatial arrangement to those in figure 4A. Unlike figure 4A, the color of the nodes in figure 4B reflects the average year of publication. Nodes displaying red tones indicate more recent academic contributions. The network shows that the nodes on the periphery of the collaboration network, many of which represent Chinese institutions, exhibit a deeper red hue; this suggests that these institutions have been quite active recently in the research area of mitophagy in PD.

**Fig. 4**

**Institutional Analysis in the Field of Mitophagy in PD.** **A** Visualization of the collaborative network among institutions using VOSviewer. This figure displays institutions with a publication count surpassing 5. The nodes, varied in color, denote different institutional clusters, and the nodes' size corresponds to the frequency of institutional involvement. **B** Institutional collaboration network map illustrating the average publication year. Nodes are colored based on the average year of publication, with redder shades indicating more recent publications and bluer shades representing older publication periods.


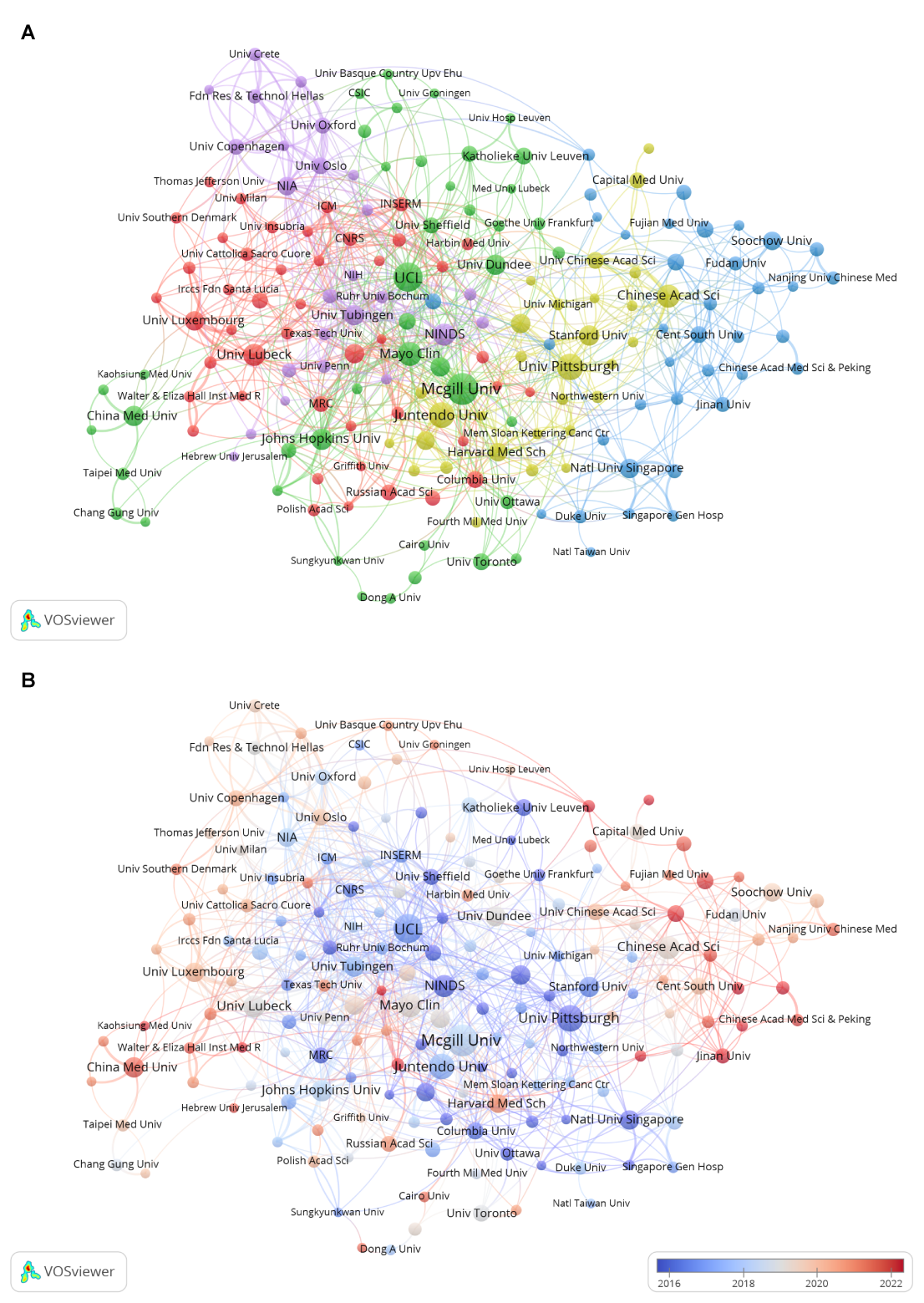


## **Analysis of journals**

**Fig. 5**

**Journal Distribution in Mitophagy Research in PD According to Bradford's Law**


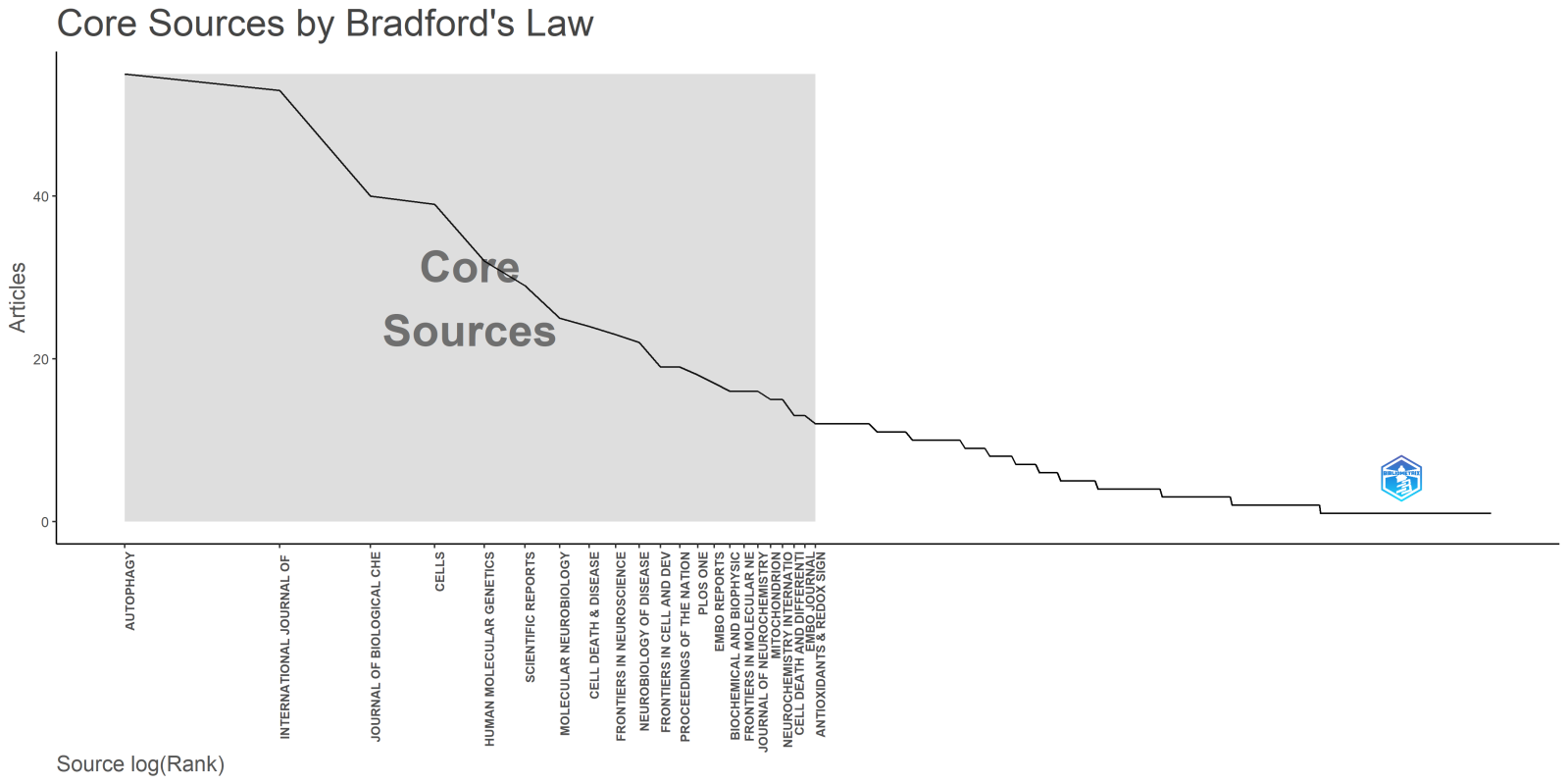


**Fig. 6**

**A** Displays the quantity of articles in core journals associated with mitophagy in PD research. **B** Illustrates the distribution of journals across Bradford's Law zones, with an emphasis on core (Zone 1), secondary (Zone 2), and peripheral (Zone 3) journals.


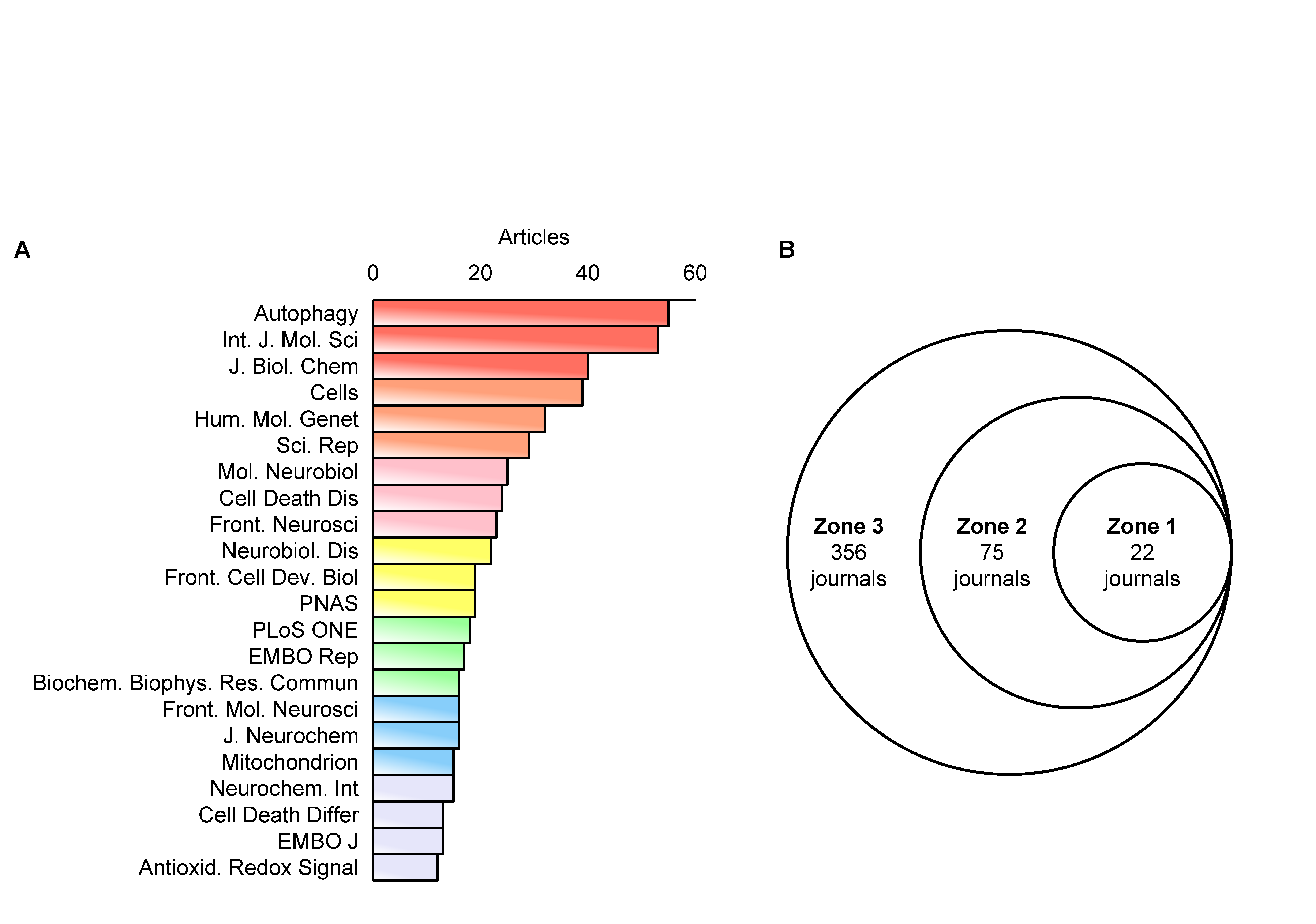


**Table 3**

Top 10 journals in terms of number of publications, corresponding IF (JCR2024) and JCR quartile

| Rank | Journal | Publications | IF（JCR2024） | JCR quartile |
| --- | --- | --- | --- | --- |
| 1 | Autophagy | 55 | 14.3 | Q1 |
| 2 | International Journal of Molecular Sciences | 53 | 4.9 | Q1 |
| 3 | Journal of Biological Chemistry | 40 | 3.9 | Q2 |
| 4 | Cells | 39 | 5.2 | Q2 |
| 5 | Human Molecular Genetics | 32 | 3.2 | Q2 |
| 6 | Scientific Reports | 29 | 3.9 | Q2 |
| 7 | Molecular Neurobiology | 25 | 4.3 | Q2 |
| 8 | Cell Death & Disease | 24 | 9.6 | Q1 |
| 9 | Frontiers in Neuroscience | 23 | 3.2 | Q2 |
| 10 | Neurobiology of Disease | 22 | 5.6 | Q1 |

When discussing the significance of journal distribution in the field of mitophagy in PD, Bradford's Law offers a crucial perspective. Bradford's Law, introduced by Samuel C. Bradford, describes a specific pattern of journal distribution within scientific literature. According to this law, if journals are sorted by the number of papers they publish on a particular subject, they can be divided into several zones, with each zone containing an equal number of papers, while the number of journals in each zone decreases geometrically, meaning a small number of core journals account for the majority of publications[32].

We utilized a bibliometric online analysis platform “bibliometirx” to identify journals in the field of mitophagy in PD. Figure 5 illustrates the distribution of core journals. The chart reveals the ranking of journals based on the logarithm of their publication count, with those in the most central area publishing a significant proportion of articles within the field. Fig. 6A displays the number of articles published in core journals in this domain. The bar chart highlights that *Autophagy* is the leading journal with 55 publications, followed by *International Journal of Molecular Sciences* (53 publications) and *Journal of Biological Chemistry* (40 publications). Other notable contributors include *Cells* and *Human Molecular Genetics.* Figure 6B provides additional detail on the number of journals in each zone. Zone 1 has 22 journals, Zone 2 has 75, and Zone 3 has 356. The 22 journals in Zone 1 are the core journals identified in Figure 5 and 6A. These journals have the highest publication volume, indicating their significant influence in the field.

Table 3 presents the top 10 journals by number of publications, co-citation frequency, impact factor (IF;JCR 2024), and JCR quartile. *Autophagy* tops the list with 55 publications and an IF of 14.3, ranking in JCR Q1.

**Fig. 7**

**Citation Network Analysis of Journals in PD Mitophagy Research.** **A** Each node represents a journal, with the size indicating the number of publications. The lines between nodes represent citation relationships. Nodes are color-coded to denote different journal clusters. **B** Node colors indicate the average normalized citation score (avg. norm. citations), with a gradient from blue (lower scores, less influence) to red (higher scores, more influence).


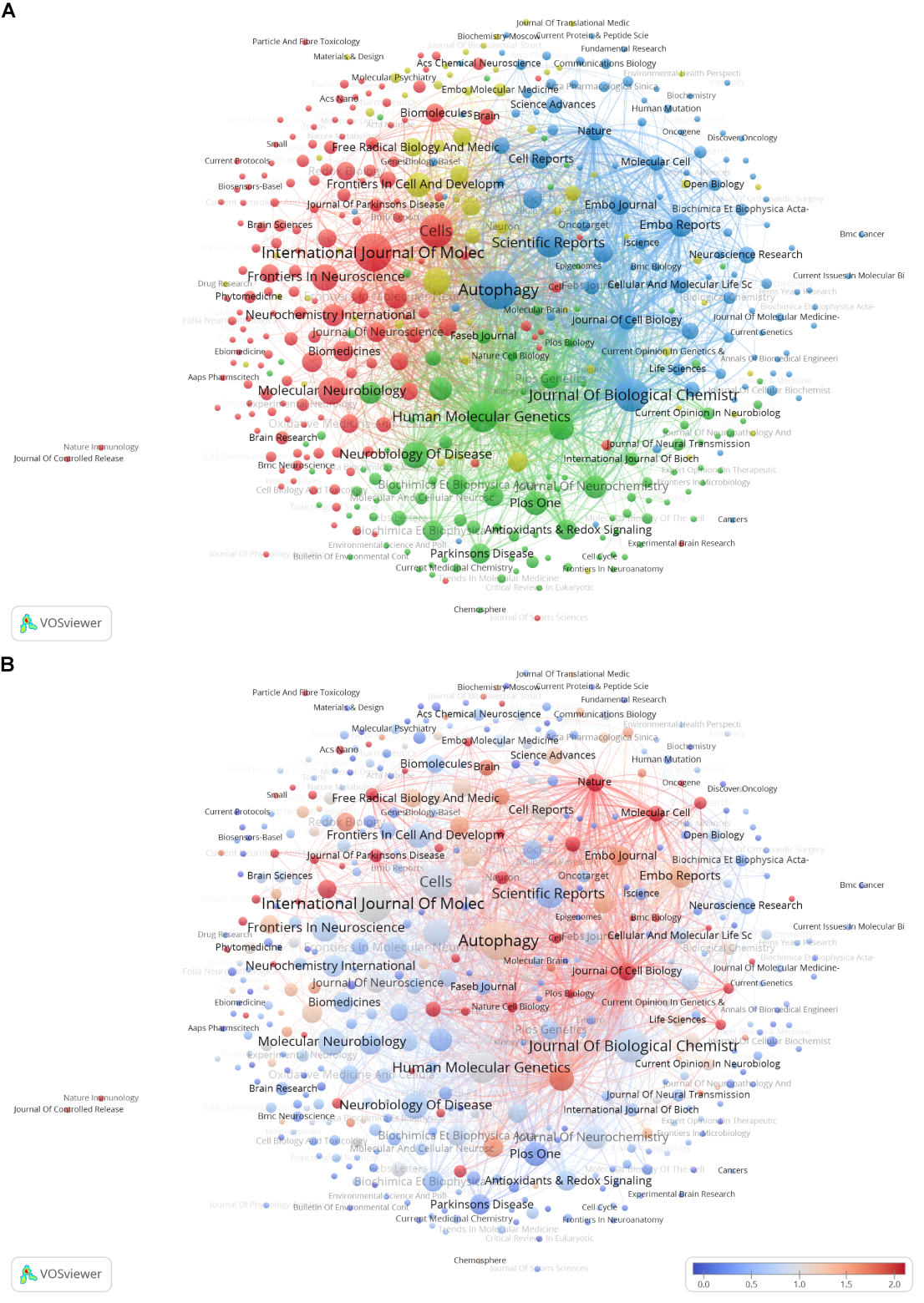


The VOSviewer visualization (figure 7A) maps journals and their interrelationships and the relatedness of items is determined based on the number of times they cite each other. The entire journal network exhibits strong and extensive connections, particularly within its central core, where journals that publish a higher volume of articles are closely interlinked. Journals are clustered into four clusters. Cluster 1 (Red) includes *International Journal of Molecular Sciences, Cells, Molecular Neurobiology, Frontiers in Neuroscience, Frontiers in Molecular Neuroscience.* Cluster 2 (Green) comprises *Human Molecular Genetics, Neurobiology of Disease, PNAS, PLOS One, Journal of Neurochemistry.* Cluster 3 (Blue) encompasses *Autophagy, Journal of Biological Chemistry, Scientific Reports, EMBO reports, EMBO Journal.* Cluster 4 (Yellow) contains *Cell Death & Disease, Frontiers in Cell and Developmental Biology, Frontiers in Aging Neuroscience, Free Radical Biology and Medicine, npj Parkinson's Disease.*

Figure 7B focuses on the average normalized citations (avg. norm. citations) metric, visualized by color intensity. Deeper red indicates a higher frequency of being cited, while bluer hues signify lower citation rates. Journals like *Nature*, *Journal of Cell Biology*, *Molecular Cell, Ageing Research Reviews* and *Neuron* appear in deep red, denoting higher average citation counts and thus prominent status and broad impact.

**Fig. 8**

**Dual-map overlay of journals displaying citation relationships.** Citing journals are positioned on the left, while cited journals are on the right, with colored paths illustrating the connections between them.


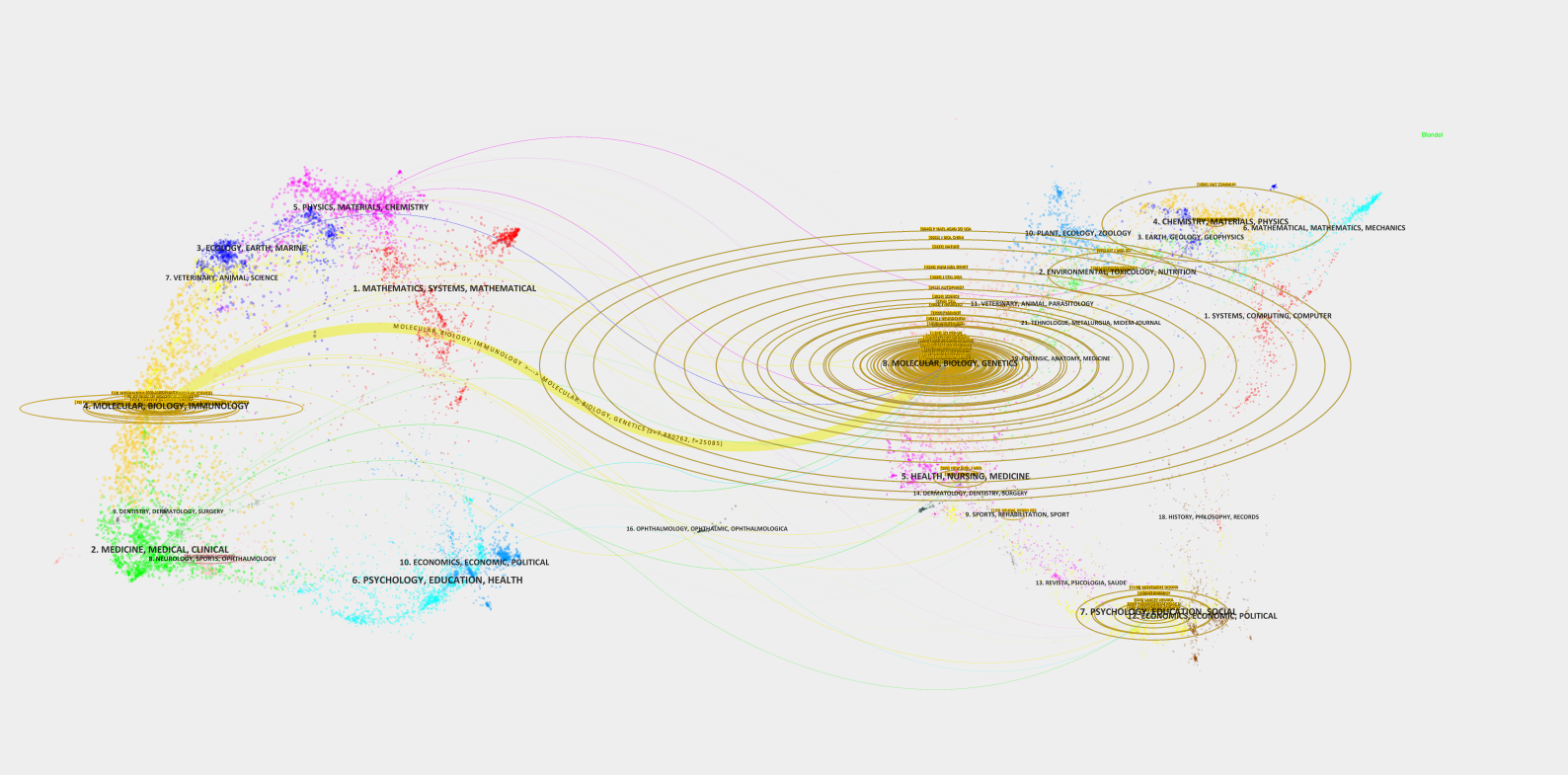


We utilized knowledge flow analysis to examine the citation and co-citation dynamics between citing and cited journals. The dual-map overlay (figure 8) illustrates the topic distribution, citation trajectory changes, and shifts in research foci across journals. The left side labels citing journals, and the right side labels cited journals. Colored curves depict knowledge flow from citing to cited journals, underscoring inter-field connectivity and influence[33]. For instance, the curve connecting "MOLECULAR BIOLOGY IMMUNOLOGY" with "MOLECULAR BIOLOGY GENETICS" suggests a significant citation relationship and knowledge flow between these two fields.

## **Analysis of authors**

## Figure 9 illustrates a comparison between the predicted distribution of document productivity among authors in the field of mitophagy in PD, based on Lotka's Law[34] (yellow area), and the actual distribution (red line). For authors with a single publication, the predicted proportion is 62%, but the observed proportion is much higher at 78.6%. This suggests that more authors than expected produce only one publication in this field. For authors publishing two or more documents, the actual proportion is lower than the predicted value, indicating that Lotka's Law may overestimate the number of highly prolific authors in this specific field.

**Fig. 9**

**Document productivity distribution among authors based on Lotka's Law**

Table 4 offers an overview of the academic influence and institutional affiliations of the top ten authors. Nobutaka Hattori tops the list with 28 publications and 3,731 citations. Richard J.Youle, with 20 publications and 12,235 citations, demonstrates exceptional influence despite fewer publications. The table also highlights authors from prestigious institutions like the University of Tübingen and McGill University, each with two representatives in the top ten, reflecting these institutions' research contributions. Authors such as Springer Wolfdieter and Edward A. Fon, with high total link strength, emerge as key collaborators. These authors and institutions play a leading role in advancing the field.

The co-authorship network map (Figure 10A) vividly illustrates the collaborative landscape among researchers. Node size reflects an author's contribution, with larger nodes indicating higher publication counts or central collaborative roles, exemplified by Hattori N and Youle RJ. Node colors differentiate clusters, highlighting groups of frequent co-authors and suggesting strong ties within specific research teams or thematic areas. Connecting lines denote co-authorship relationships, with closely positioned nodes, such as Fon EA and Jean-Francois Trempe, indicating potential research partnerships. Figure 10B uses average normalized citations as the metric. The visualization employs a color gradient, with darker red shades indicating higher academic impact. Prominent authors such as Youle RJ, Springer W, Hattori N and Fon EA are highlighted, reflecting their influential contributions and frequent citations. The map also shows small, emerging networks. Though currently limited, they could signal new directions and grow into larger clusters. Monitoring them is crucial for future breakthroughs.

Figure 11A focuses authors with at least eight publications, excluding isolated nodes. It highlights active collaborators, with clusters indicating closely working groups and lines showing collaborative ties. Central authors serve as key connectors. This map provides a clear view of leading scholars' collaborative relationships. Figure 11B shows the same network with nodes colored by average publication year. Blue to red gradients indicate older to newer publications. Red nodes highlight recent activity, while blue nodes show older contributions. Notably, the teams of Springer W and Fabienne C. Fiesel, as well as the team of Fon EA, are highlighted with red nodes, indicating their recent contributions.

**Table 4**

Top 10 authors in terms of number of publications

| Rank | Author | Number of publication | Ciations | total link strength | Institutions |
| --- | --- | --- | --- | --- | --- |
| 1 | Hattori Nobutaka | 28 | 3731 | 229 | Juntendo Univ |
| 2 | Edward A. Fon | 26 | 3252 | 184 | McGill Univ |
| 3 | Springer Wolfdieter | 26 | 3918 | 273 | Mayo Clinic |
| 4 | Fabienne C. Fiesel | 23 | 3739 | 256 | Mayo Clinic |
| 5 | Richard J. Youle | 20 | 12235 | 101 | NINDS |
| 6 | Charleen T. Chu | 19 | 1585 | 55 | Univ Pittsburgh |
| 7 | Christine Klein | 19 | 1544 | 188 | Univ Lubeck |
| 8 | Noriyuki Matsuda | 17 | 3280 | 121 | Tokyo Metropolitan Inst Med Sc |
| 9 | Anne Gruenewald | 16 | 1744 | 171 | Univ Luxembourg |
| 10 | Jean-Francois Trempe | 16 | 829 | 120 | McGill Univ |

**Fig. 10**

**Authorship Analysis of Mitophagy Research in PD.** **A** This figure includes the top 1000 authors out of 7428 based on total link strength, excluding those not connected to other nodes, resulting in a final network of 802 authors. The nodes, differentiated by color, represent authors within various clusters, with node size reflecting the frequency of their publications. **B** Network map highlighting key authors, with nodes colored by average normalized citations (avg. norm. citations). Redder nodes indicate higher academic influence.


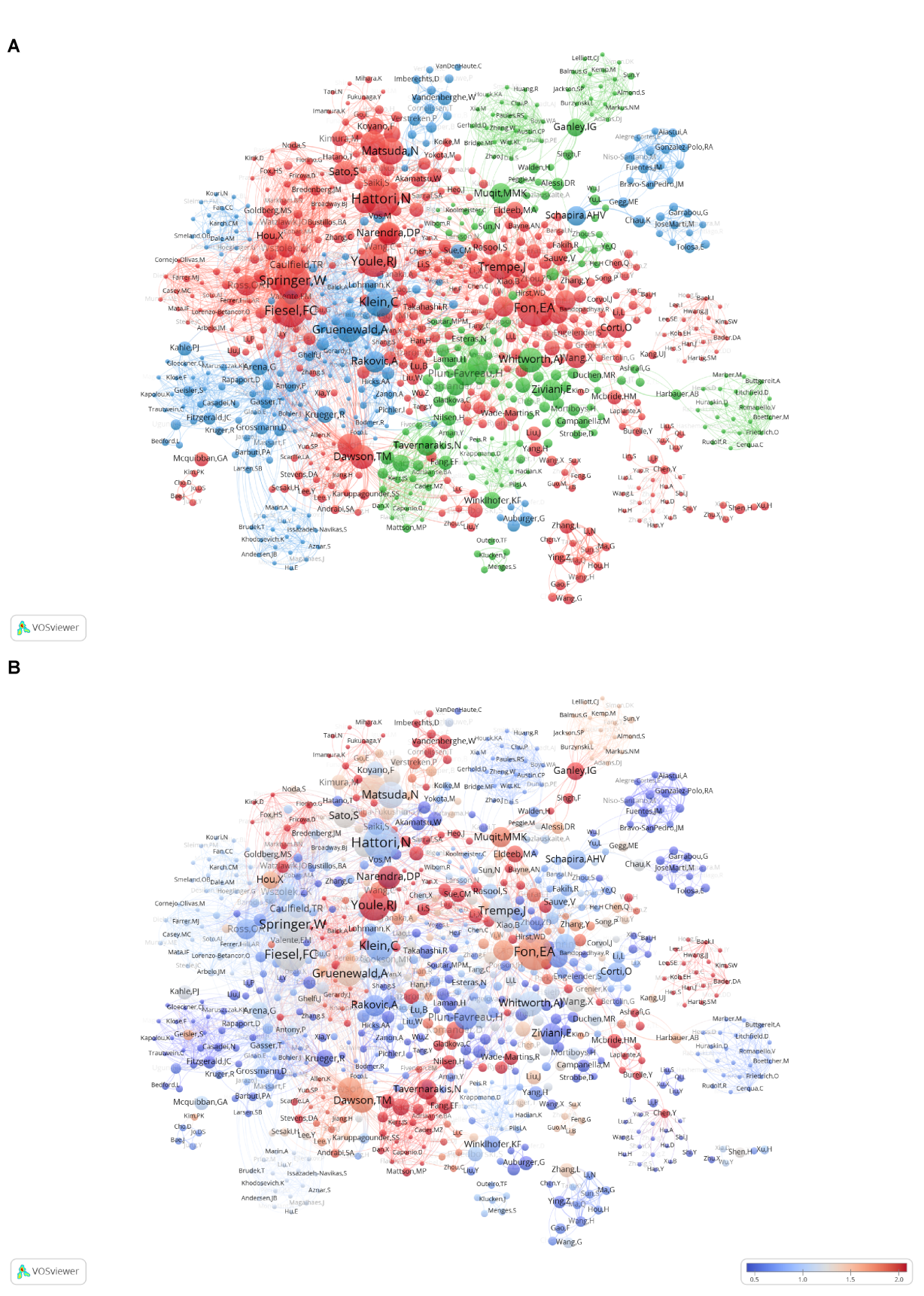


**Fig. 11**

**Collaboration network of key authors with a minimum of 8 publications in mitophagy research. A** This network includes authors who have published 8 or more papers, with 41 authors remaining after excluding those without connections to other nodes. The nodes, differentiated by color, represent authors within various clusters, with node size reflecting the frequency of their publications. **B** Node colors show the average publication year. Redder nodes mean more recent activity, bluer nodes indicate older work. This helps spot active researchers and groups in the field.


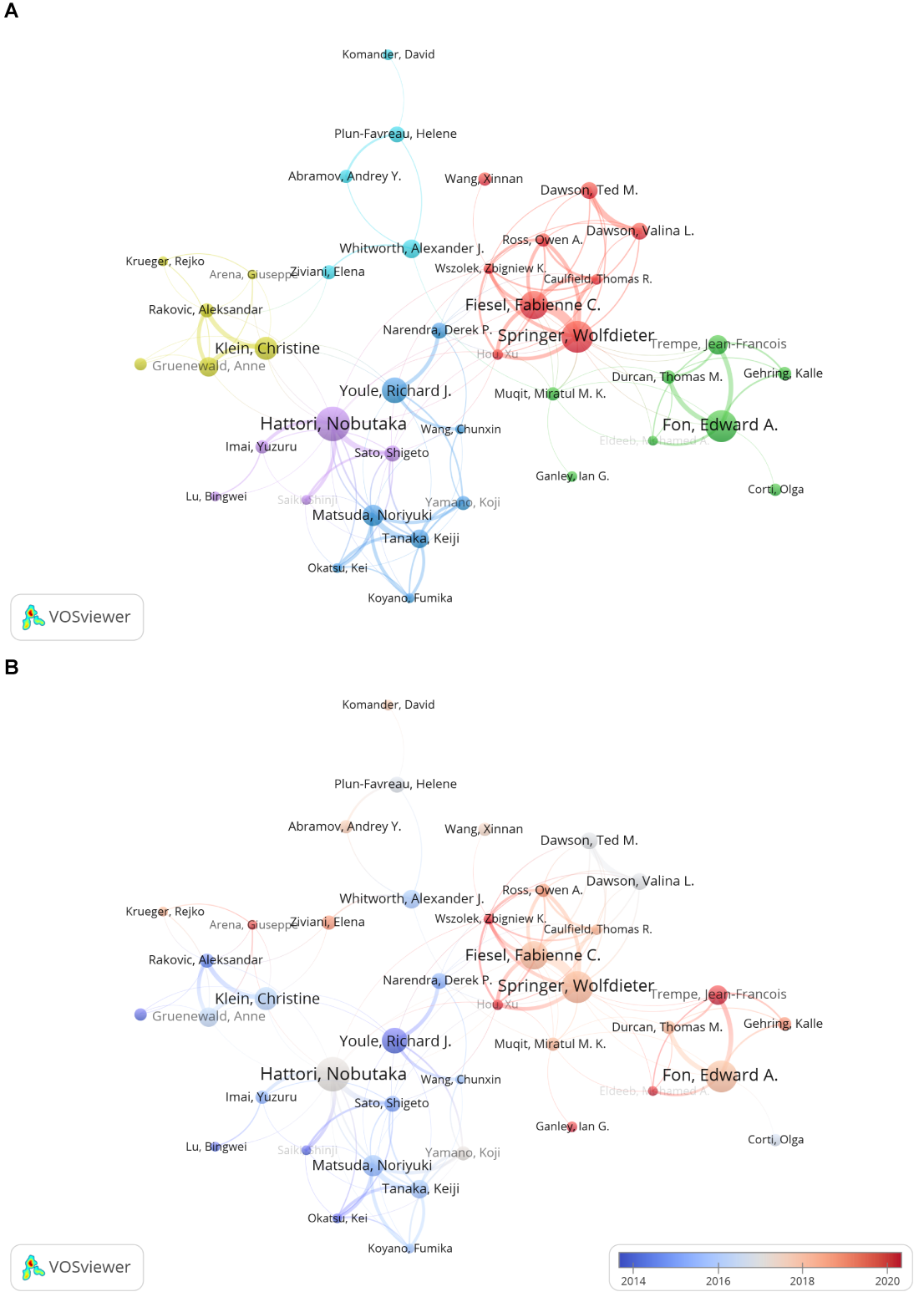


**Analysis of keyword and topic**

**Table 5**

Top 20 keywords in mitophagy research in PD with occurrences and total link strength

| Rank | Keyword | Occurrences | Total link strength |
| --- | --- | --- | --- |
| 1 | mitophagy | 969 | 9948 |
| 2 | pink1 | 494 | 5254 |
| 3 | autophagy | 484 | 5068 |
| 4 | parkinson’s disease | 467 | 5135 |
| 5 | mitochondria | 432 | 4810 |
| 6 | oxidative stress | 390 | 4215 |
| 7 | parkinson disease | 382 | 3878 |
| 8 | parkin | 357 | 3989 |
| 9 | α-synuclein | 319 | 3492 |
| 10 | mutations | 270 | 2661 |
| 11 | mitochondrial dysfunction | 230 | 2375 |
| 12 | neurodegeneration | 218 | 2419 |
| 13 | ubiquitin | 189 | 2102 |
| 14 | disease | 182 | 1814 |
| 15 | degradation | 180 | 1852 |
| 16 | dysfunction | 176 | 1674 |
| 17 | activation | 158 | 1538 |
| 18 | damaged mitochondria | 144 | 1639 |
| 19 | protein | 142 | 1407 |
| 20 | cell-death | 136 | 1437 |

Keywords offer a snapshot of key research themes. Table 5 lists the top 20 keywords by frequency. The most common are “mitophagy” (969), “pink1” (494), “autophagy” (484), “parkinson’s disease” (467), and “mitochondria” (432). The prominence of “pink1” and “parkin” underscores their critical roles in mitophagy and mitochondrial quality control in the context of PD. Additionally, other notable keywords such as “oxidative stress” (390), “α-synuclein” (319), and “mutations” (270) highlight the multifaceted aspects of the disease pathology, including mitochondrial dysfunction, oxidative stress, and genetic mutations. Collectively, these keywords reflect the complex interplay between mitophagy, mitochondrial health, and neurodegeneration in PD.

A co-occurrence network diagram of keywords visualized in VOSviewer (Figure 12) reveals seven major clusters based on research themes. The first cluster addresses the multifactorial pathogenesis of PD, including oxidative stress, mitochondrial dysfunction, and neuroinflammation. The second cluster focuses on the PINK1/Parkin signaling pathway and its role in mitophagy and disease pathology. The third cluster examines the interplay between mitochondrial dysfunction and protein aggregation. The fourth cluster highlights the relationship between calcium signaling and mitochondrial damage. The fifth cluster explores mitophagy and oxidative stress mechanisms and their therapeutic potential. The sixth cluster investigates genetic mechanisms and potential therapeutic targets. The seventh cluster delves into protein aggregation and autophagy mechanisms.

**Fig. 12**

**Co-occurrence network of keywords in the study of mitophagy in PD.** The network in VOSviewer shows co-occurrence relationships among keywords, organized into seven color-coded clusters from right to left: red (Cluster 1), green (Cluster 2), blue (Cluster 3), yellow (Cluster 4), purple (Cluster 5), light blue (Cluster 6), and orange (Cluster 7).


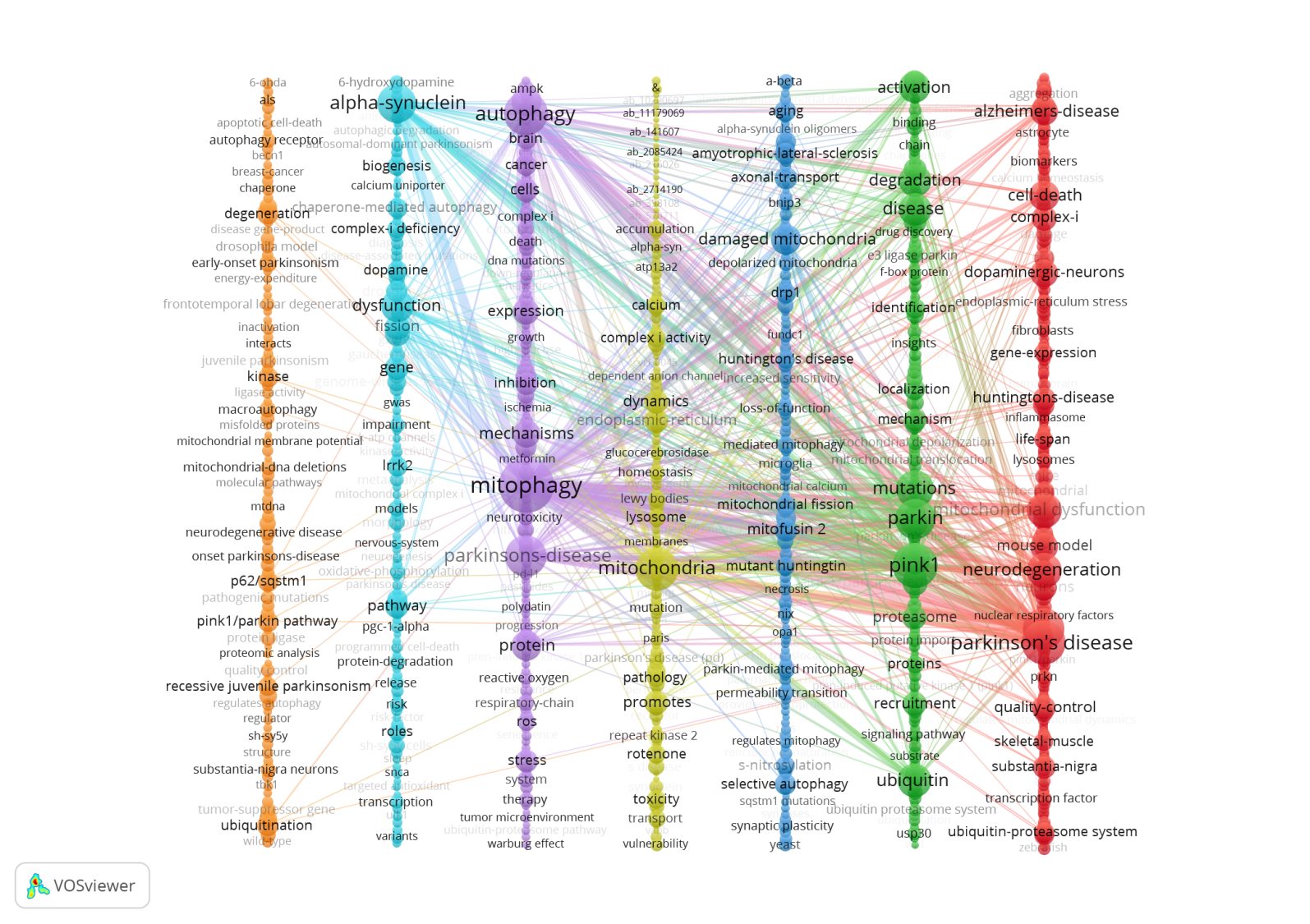


**Fig. 13**

**Trend analysis of key terms in mitophagy research in PD**


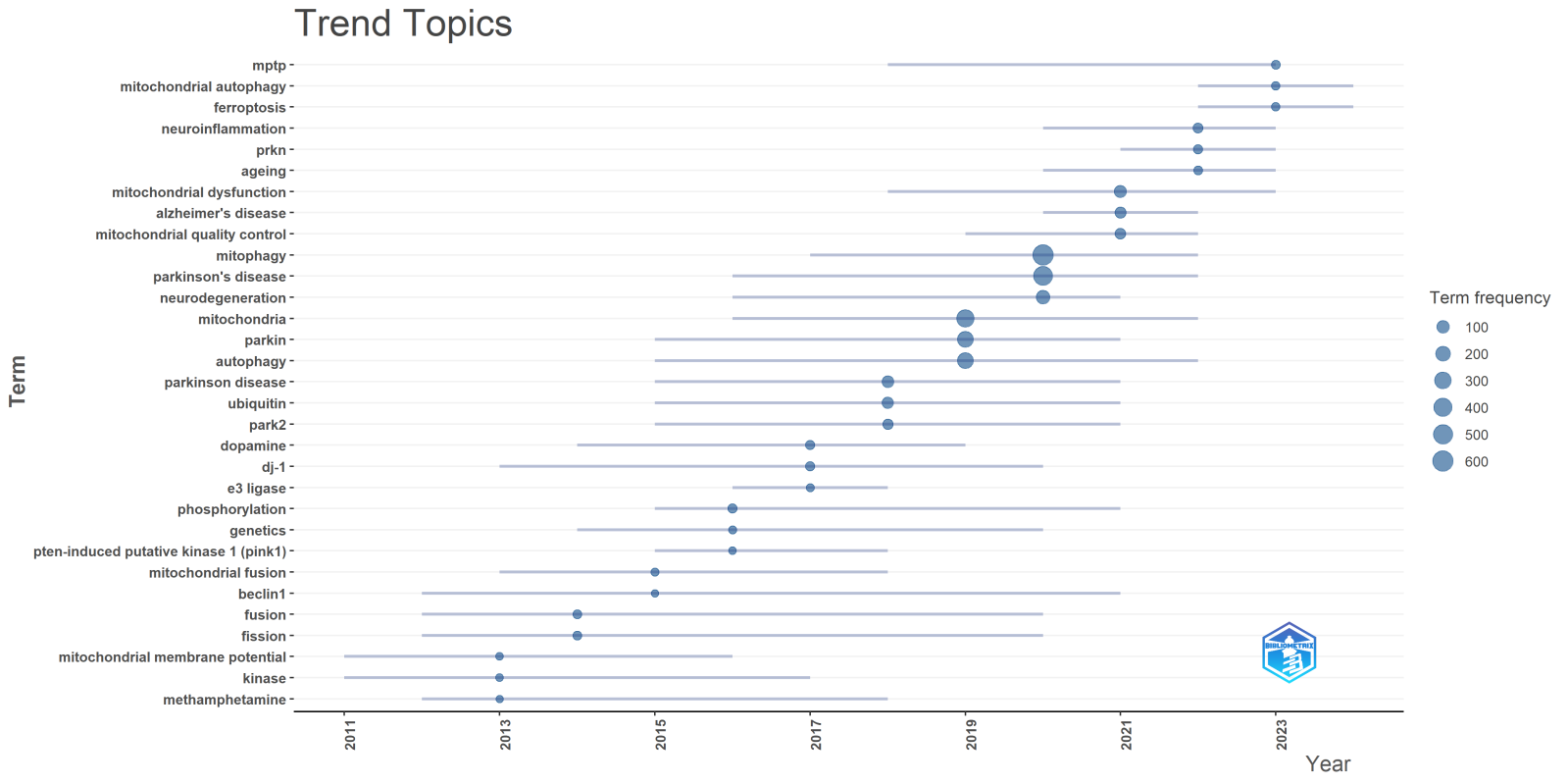


The trend topic chart (figure 13) generated by Bibliometrix illustrates the evolution and popularity of research themes. The size of each dot reflects the prevalence of a specific research focus over time. Terms like "neuroinflammation" and "ferroptosis" show increasing trends, highlighting emerging areas of interest. This reflects growing recognition of the complex interplay between mitochondrial dysfunction and other cellular processes, such as inflammation and regulated cell death, in PD pathogenesis. The increasing frequency of these terms may indicate a shift in research focus towards understanding the multifaceted mechanisms underlying neurodegenerative processes and identifying potential therapeutic targets.

**Fig. 14**

**Heatmap analysis of keywords.** **A** Annual heatmap from 2007 to 2024. The annual heat value of each keyword is obtained by dividing the number of citations in that year by the total number of citations in that year. **B** Keyword relevance heatmap. Keywords with high popularity in similar time periods are clustered into one category and marked with different colors.


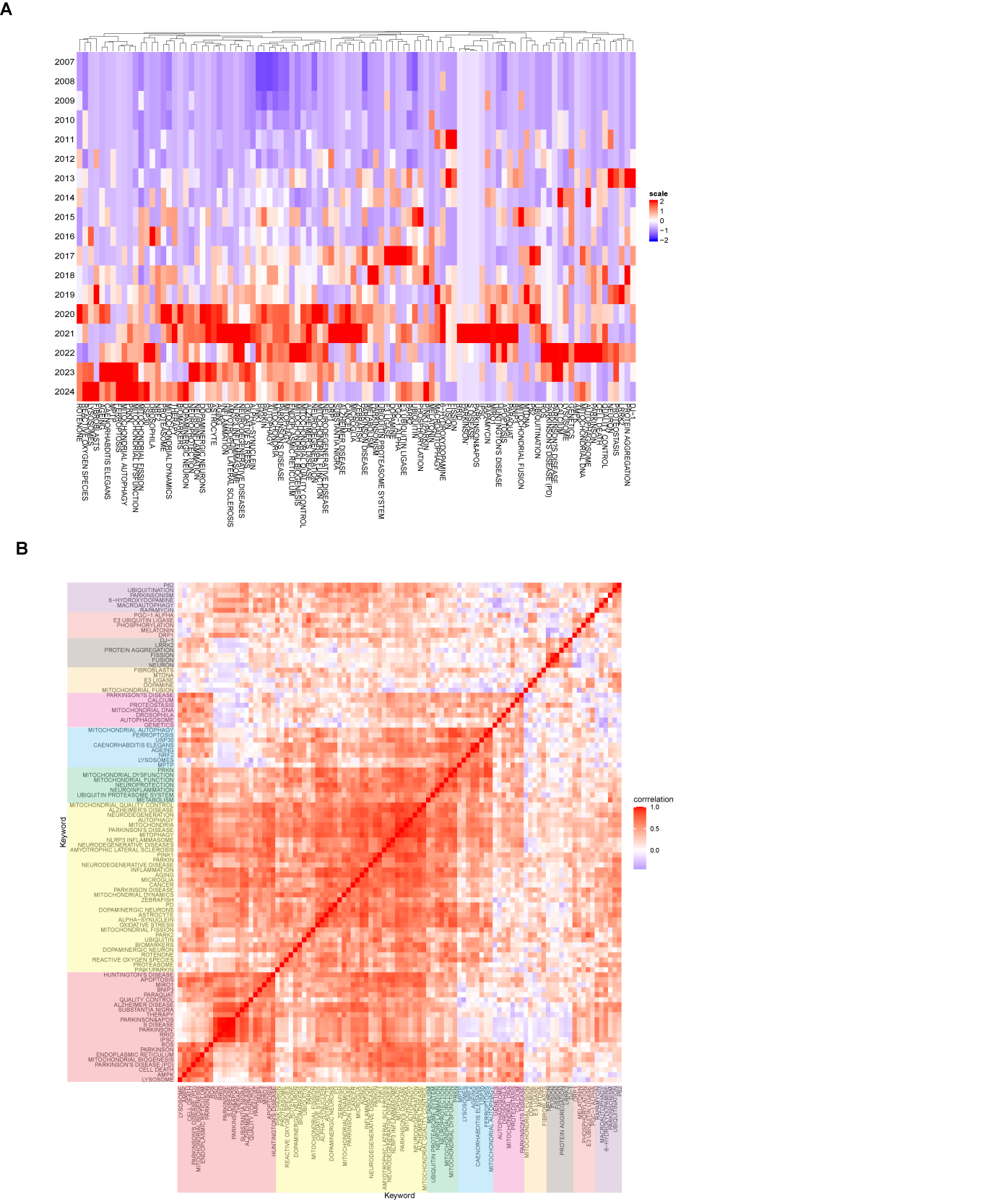


Figure 14A presents an annual heatmap analysis of research keywords from 2007 to 2024, visualizing the annual popularity of each keyword. This popularity is calculated by dividing the number of citations for that keyword in a specific year by the total number of citations for that year. In the last two years, keywords such as "pink1/parkin," "neuroinflammation," "neuroprotection," "dopaminergic neuron," "usp30," "mitochondrial fission," "mitochondrial dysfunction," "prkn," "ferroptosis," "mptp," "caenorhabditis elegans," "ageing," "fibroblasts," "lysosomes," and "reactive oxygen species" have become hot topics. Figure 14B illustrates a high degree of correlation among keywords within the research field of mitophagy in PD. This high correlation suggests that the research topics in this field are closely interconnected and likely revolve around several core concepts or issues. This could imply that researchers are generally focused on similar scientific questions and employ similar theoretical frameworks and methodologies.Furthermore, this high degree of correlation may indicate that the research trends in this field are relatively stable, with researchers consistently focusing on certain hot topics rather than frequently shifting their research focus. This could reflect the maturity of the field, where some key issues have gained widespread ecognition and in-depth study. However, despite the generally high correlation between most keywords, there may still be some keywords or themes with lower correlation. These keywords with lower correlation might represent emerging areas of research or potential research gaps that warrant further exploration.

# **Highly cited publications**

**Table 6**

Top 15 high-cited publications related to mitophagy in PD

| Rank | Authors | Article Title | Source Title | Document Type | Times Cited | Publication Year | DOI |
| --- | --- | --- | --- | --- | --- | --- | --- |
| 1 | Youle, RJ et al. | Mechanisms of mitophagy[22] | NATURE REVIEWS MOLECULAR CELL BIOLOGY | Review | 2487 | 2011 | https://doi.org/10.1038/nrm3028 |
| 2 | Geisler, S et al. | PINK1/Parkin-mediated mitophagy is dependent on VDAC1 and p62/SQSTM1[35] | NATURE CELL BIOLOGY | Article | 2218 | 2010 | https://doi.org/10.1371/journal.pbio.1000298 |
| 3 | Narendra, DP et al. | PINK1 Is Selectively Stabilized on Impaired Mitochondria to Activate Parkin[36] | PLOS BIOLOGY | Article | 2204 | 2010 | https://doi.org/10.1371/journal.pbio.1000298 |
| 4 | Hou, YJ et al. | Ageing as a risk factor for neurodegenerative disease[37] | NATURE REVIEWS NEUROLOGY | Review | 1737 | 2019 | https://doi.org/10.1038/s41582-019-0244-7 |
| 5 | Pickrell, AM et al. | The Roles of PINK1, Parkin, and Mitochondrial Fidelity in Parkinson's Disease[38] | NEURON | Review | 1558 | 2015 | https://doi.org/10.1016/j.neuron.2014.12.007 |
| 6 | Matsuda, N et al. | PINK1 stabilized by mitochondrial depolarization recruits Parkin to damaged mitochondria and activates latent Parkin for mitophagy[19] | JOURNAL OF CELL BIOLOGY | Article | 1494 | 2010 | https://doi.org/10.1083/jcb.200910140 |
| 7 | Ashrafi, G et al. | The pathways of mitophagy for quality control and clearance of mitochondria[39] | CELL DEATH AND DIFFERENTIATION | Review | 1306 | 2013 | https://doi.org/10.1038/cdd.2012.81 |
| 8 | Dias, V et al. | The Role of Oxidative Stress in Parkinson's Disease[40] | JOURNAL OF PARKINSONS DISEASE | Review | 1303 | 2013 | https://doi.org/10.3233/JPD-130230 |
| 9 | Vives-Bauza, C et al. | PINK1-dependent recruitment of Parkin to mitochondria in mitophagy[41] | PROCEEDINGS OF THE NATIONAL ACADEMY OF SCIENCES OF THE UNITED STATES OF AMERICA | Article | 1295 | 2010 | https://doi.org/10.1073/pnas.0911187107 |
| 10 | Chen, HC et al. | Mitochondrial dynamics-fusion, fission, movement, and mitophagy-in neurodegenerative diseases[42] | HUMAN MOLECULAR GENETICS | Review | 1135 | 2009 | https://doi.org/10.1093/hmg/ddp326 |
| 11 | Tanaka, A et al. | Proteasome and p97 mediate mitophagy and degradation of mitofusins induced by Parkin[43] | JOURNAL OF CELL BIOLOGY | Article | 1088 | 2010 | https://doi.org/10.1083/jcb.201007013 |
| 12 | Fang, EF et al. | Mitophagy inhibits amyloid-β and tau pathology and reverses cognitive deficits in models of Alzheimer's disease[44] | NATURE NEUROSCIENCE | Article | 1082 | 2019 | https://doi.org/10.1038/s41593-018-0332-9 |
| 13 | Chen, Y et al. | PINK1-Phosphorylated Mitofusin 2 Is a Parkin Receptor for Culling Damaged Mitochondria[45] | SCIENCE | Article | 1019 | 2013 | https://doi.org/10.1126/science.1231031 |
| 14 | Jin, SM et al. | Mitochondrial membrane potential regulates PINK1 import and proteolytic destabilization by PARL[46] | JOURNAL OF CELL BIOLOGY | Article | 1017 | 2010 | https://doi.org/10.1083/jcb.201008084 |
| 15 | Wang, XN et al. | PINK1 and Parkin Target Miro for Phosphorylation and Degradation to Arrest Mitochondrial Motility[47] | CELL | Article | 910 | 2011 | https://doi.org/10.1016/j.cell.2011.10.018 |

# **Highly cited reference analysis**

****Table 7****

**Top 10 highly cited references on Mitophagy in** PD**: Insights from CiteSpace LBY:5**

| Rank | Article Title | Article type | Source | Authors | Year | Cited | IF（JCR2024） | JCR quartile | DOI |
| --- | --- | --- | --- | --- | --- | --- | --- | --- | --- |
| 1 | PINK1/Parkin-mediated mitophagy is dependent on VDAC1 and p62/SQSTM1[35] | Article | Nature Cell Biology | Geisler S | 2010 | 218 | 17.3 | Q1 | https://doi.org/10.1038/ncb2012 |
| 2 | PINK1 is selectively stabilized on impaired mitochondria to activate Parkin[36] | Article | PLoS Biology | Narendra DP | 2010 | 211 | 7.2 | Q1 | https://doi.org/10.1371/journal.pbio.1000298 |
| 3 | PINK1-dependent recruitment of Parkin to mitochondria in mitophagy[41] | Article | Proceedings of the National Academy of Sciences | Vives-Bauza C | 2010 | 176 | 9.4 | Q1 | https://doi.org/10.1073/pnas.0911187107 |
| 4 | The roles of PINK1, parkin, and mitochondrial fidelity in Parkinson's disease[38] | Review | Neuron | Pickrell AM | 2015 | 169 | 14.7 | Q1 | https://doi.org/10.1016/j.neuron.2014.12.007 |
| 5 | The ubiquitin kinase PINK1 recruits autophagy receptors to induce mitophagy[48] | Article | Nature | Lazarou M | 2015 | 161 | 50.5 | Q1 | https://doi.org/10.1038/nature14893 |
| 6 | PINK1 stabilized by mitochondrial depolarization recruits Parkin to damaged mitochondria and activates latent Parkin for mitophagy[19] | Article | The Journal of Cell Biology | Matsuda N | 2010 | 160 | 7.4 | Q1 | https://doi.org/10.1083/jcb.200910140 |
| 7 | Ubiquitin is phosphorylated by PINK1 to activate parkin[49] | Article | Nature | Koyano F | 2014 | 147 | 50.5 | Q1 | https://doi.org/10.1038/nature13392 |
| 8 | Parkin is recruited selectively to impaired mitochondria and promotes their autophagy[50] | Article | The Journal of Cell Biology | Narendra DP | 2008 | 147 | 7.4 | Q1 | https://doi.org/10.1083/jcb.200809125 |
| 9 | PINK1 phosphorylates ubiquitin to activate Parkin E3 ubiquitin ligase activity[51] | Article | The Journal of Cell Biology | Kane LA | 2014 | 133 | 7.4 | Q1 | https://doi.org/10.1083/jcb.201402104 |
| 10 | Mitophagy inhibits amyloid-β and tau pathology and reverses cognitive deficits in models of Alzheimer's disease[44] | Article | Nature Neuroscience | Fang EF | 2019 | 125 | 21.2 | Q1 | https://doi.org/10.1038/s41593-018-0332-9 |

Table 7 presents the top 10 most cited references. These references were identified using CiteSpace software, with the "Look Back Year (LBY)" parameter set at 5 years. This means that CiteSpace considered the citation status of each paper within 5 years after its publication. The purpose of this setting is to focus more precisely on recent research advancements and streamline the analysis network. By limiting the analysis to the most recent 5 years, we aim to identify papers that have garnered significant attention shortly after their publication, rather than those that have accumulated citations over a longer period. This approach helps to more accurately reflect the current research landscape and identify key papers that are driving recent trends. However, it is also worth noting that this setting may exclude some early foundational articles in the field. For example, the seminal work by Valente et al. on the identification of mutations in PINK1 as a cause of hereditary early-onset PD is a foundational study in this field[9]. Similarly, Clark et al. demonstrated the role of Drosophila PINK1 in mitochondrial function and its genetic interaction with parkin[52], while Park et al. further elucidated the mitochondrial dysfunction in Drosophila PINK1 mutants and how it can be complemented by parkin[53]. These early studies laid the groundwork for understanding the role of PINK1 in PD but may not be included in our analysis due to the 5-year LBY parameter. Despite this potential limitation, the current analysis still provides valuable insights into the most impactful recent research in the field.

Among these highly cited papers, 90% are original research articles. A significant portion of these highly cited publications overlaps with the previously mentioned top 15 highly cited publications (see Table 6), highlighting their importance and representativeness.

**Fig. 15**

**Reference analysis in the context of mitophagy in PD.** **A** Visualization of the reference network in CiteSpace. The size of each node corresponds to the frequency with which the respective article is co-cited. The network map highlights the collaborative ties among authors, with lines indicating co-citation relationships. The color gradient ranging from purple to red signifies the publication years, with purple representing earlier publications and red indicating more recent ones. **B** References are clustered based on title similarity, with each cluster color-coded and labeled with numbers and themes, including topics such as #0 selective autophagy, #1 pink1-associated Parkinson's disease, #2 intracellular organelle, #3 e3 ubiquitin ligase parkin, #4 autophagic cell stress, #5 neuronal damage, #6 targeting mitophagy, #7 mitochondrial dynamics, #8 sqstm1 cooperate, #9 mitochondrial fission, #10 small n-terminal tag, #11 cytosolic pink1, and #12 mitochondrial morphology, #13 mitophagy in neurodegenerative diseases.


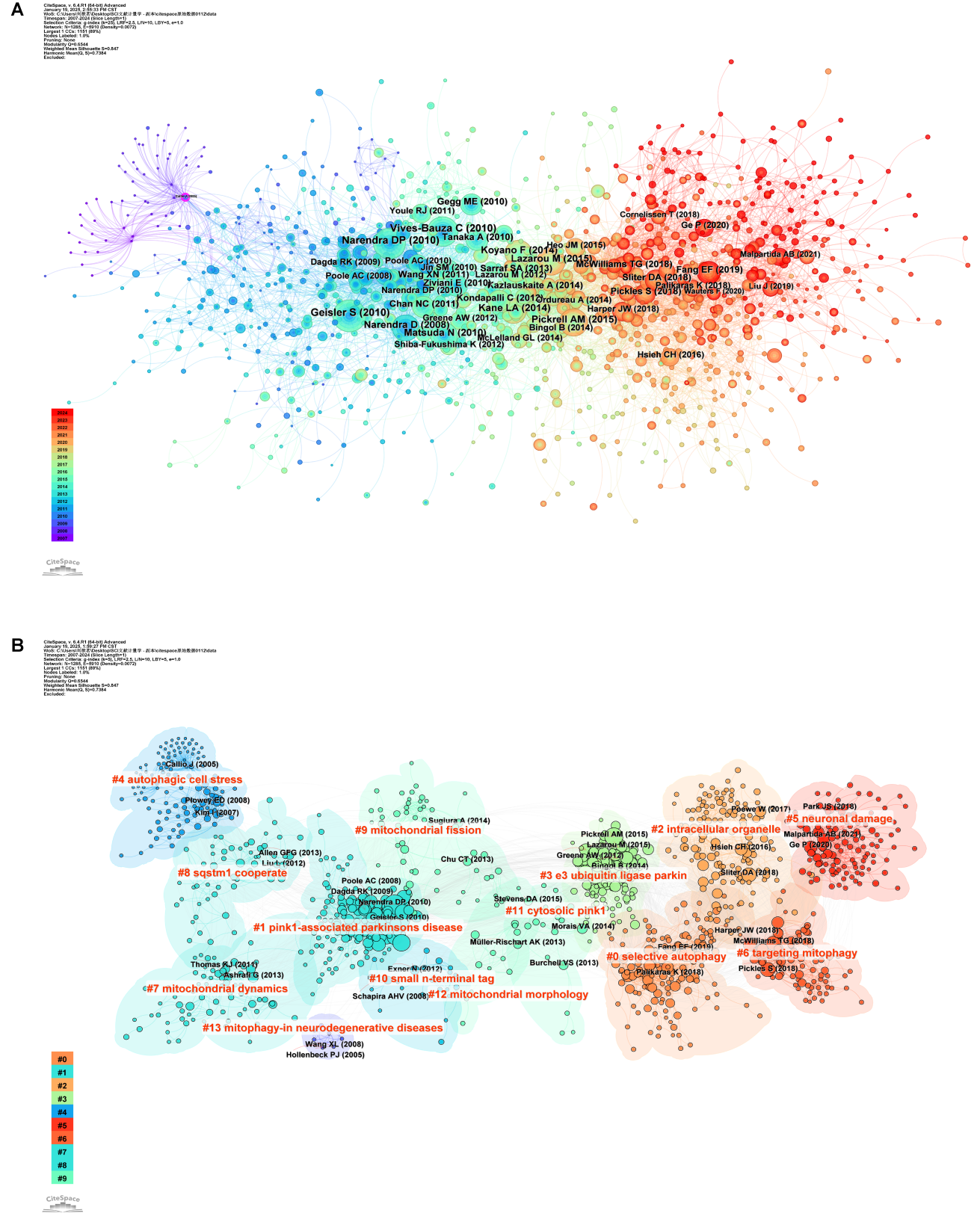


**Fig. 16**

**A** Dependency relationships among co-cited literature clusters in the research on mitophagy in PD, generated by displaying the top 50% of cluster dependency paths. The direction of the arrows between clusters indicates the direction of influence, such as an arrow pointing from Cluster B to Cluster A signifies that the development of Cluster A is influenced by Cluster B. **B** The top 25 references with the strongest citation bursts.


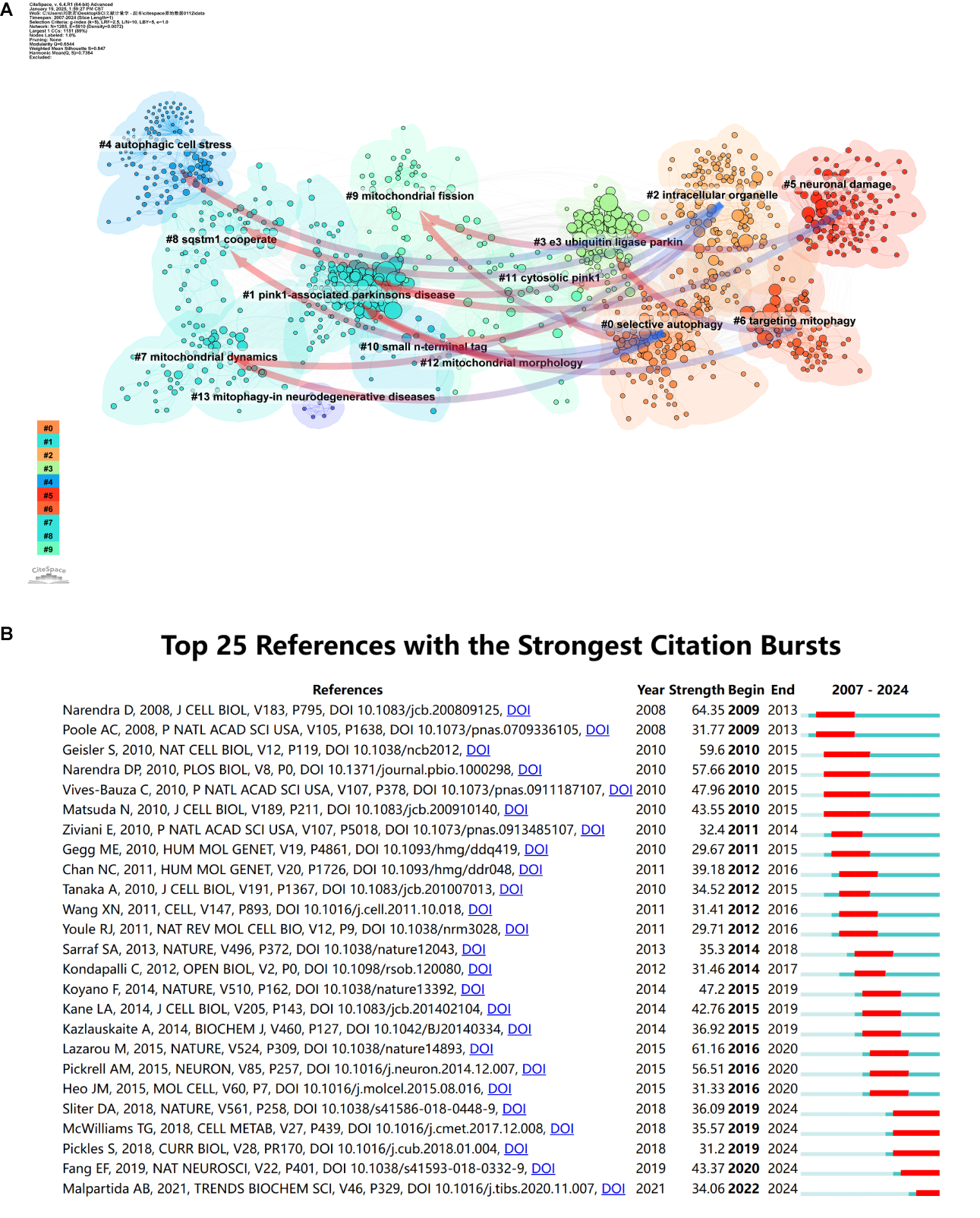


Article co-citation analysis reveals the thematic, methodological, or theoretical connections between papers by tracking their co-citation frequency. In CiteSpace (with LBY=5, see figure 15A and 15B), each node represents a paper, with its size proportional to co-citation frequency (larger nodes indicate more co-citations) and color mapping to the "citation year" (from cold to warm tones for early to recent citations). Given the LBY=5 setting, only citations within 5 years post-publication are counted, thus the map reflects short-term academic impact. The large blue nodes in the center of the map (e.g.,papers from 2009–2014) indicate that these papers were highly co-cited within five years of publication. Despite their earlier publication dates, their conclusions or methods continue to influence subsequent research, suggesting they are core theories or classic findings in the field and remain valuable references. Conversely, the large warm-toned (red) nodes on the right represent recently published papers that have been quickly cited in a short period. Their research directions or innovations may signal emerging hotspots or future trends in the field and deserve close attention.

Figure 16A shows the results of a bibliometric analysis using CiteSpace, focusing on cluster dependencies and highlighting the top 50% of these paths. The visualization reveals how one cluster influences another, with arrow directions indicating the flow of influence. For example, an arrow from Cluster B to Cluster A means that Cluster A's development is influenced by Cluster B. Firstly, cluster #0, "Selective Autophagy" is influenced by several other clusters, including "Pink1-associated Parkinson's Disease", "Mitochondrial Dynamics", "SQSTM1 Cooperation", "Mitochondrial Fission", "Cytosolic Pink1", and "Mitochondrial Morphology". This indicates that progress in selective autophagy research is closely tied to these areas, likely intersecting in mechanisms, disease associations, and therapeutic strategies. Secondly, Cluster#2, "Intracellular Organelle" is influenced by several clusters, including "Pink1-associated Parkinson's Disease", "Mitochondrial Dynamics", "SQSTM1 Cooperation", "Mitochondrial Fission" and "Small N-terminal Tag". This highlights the significant interplay between intracellular organelle research and studies on mitochondrial function, morphology, and protein tags. Additionally, both Cluster #5, "Neuronal Damage" and Cluster #6, "Targeting Mitophagy" are influenced by "Pink1-associated Parkinson's Disease" and "E3 Ubiquitin Ligase Parkin." This underscores the importance of Pink1 and Parkin research in understanding neuronal damage and mitophagy targeting.

Figure 16B highlights the top 25 references with the strongest citation bursts. The first two bursts occurred in 2009, with papers titled "Parkin is recruited selectively to impaired mitochondria and promotes their autophagy"[50] and "The PINK1/Parkin pathway regulates mitochondrial morphology"[54]. Notably, the paper by Narendra et al. has the strongest burst (strength=64.35) and its burst duration lasted until 2013. Another high-burst paper is "The ubiquitin kinase PINK1 recruits autophagy receptors to induce mitophagy" by Lazarou et al. (strength=61.16)[48]. The data shows that papers published in 2010 caused the most citation bursts (seven in total), indicating a surge in related research activities.

# **Discussion**

**Early pioneering studies and their Impact on mitophagy research in PD**

Our study identified relevant literature starting from 2007 by using the search terms TS=(Parkinson* OR PD) AND TS=(mitophagy OR "mitochondrial autophagy"). It is important to note that foundational studies in the field of mitophagy date back to the 1990s. For instance, the initial reports of mutations in the PRKN and PINK1 genes[9][10]. provided crucial insights into the genetics of PD. Additionally, research in Drosophila melanogaster elucidated the functional interaction between PRKN and PINK1 in a common pathway[52][55], which is now recognized as a key component of mitophagy. These early studies laid the groundwork for subsequent research on mitophagy and have significantly influenced the development of the field. Although our main analysis focuses on research trends from 2007 onwards to capture recent developments, the seminal contributions of these early studies must be acknowledged.

**Research trends and future prospects**

In this study, we utilized a nonlinear fitting curve to elucidate the annual publication growth trajectory of mitophagy research in PD. This trend aligns with the theory of scientific development proposed by Derek J. de Solla Price[56], which posits that the volume of scientific literature in a particular field will initially exhibit exponential growth before eventually plateauing. Notably, the inflection point of this growth pattern can be traced back to around 2010, a period during which key breakthrough studies laid the foundation for the subsequent establishment of paradigms. For instance, Narendra et al. revealed the mechanism by which PINK1 accumulates on damaged mitochondria and how this accumulation triggers the recruitment of Parkin and mitophagy[36]. Additionally, the team of Vives-Bauza elucidated how PINK1 and Parkin jointly regulate mitochondrial transport and aggregation, facilitating autophagic degradation in the perinuclear region[41]. These studies provided the molecular framework for the subsequent rapid growth phase of publications. The current surge in publication numbers indicates that the field has established a dominant paradigm and is in the application phase, characterized by the rapid expansion of knowledge and the widespread dissemination of established theories and methods. This period has benefited from recent in-depth research on PD-related mitochondrial dysfunction, such as the discovery of the interaction between α-synuclein and mitochondria[57], as well as technological advancements like CRISPR gene editing[58]. Moreover, policy funding has also been a powerful driving force behind the expansion of this field. By using the search term ("parkinson's disease” AND “mitophagy") to access the official NIH RePORTER database (https://reporter.nih.gov), we obtained funding data from 2007 to 2024. The initial funding in 2007 was 5.8 million dollars, which increased to 25 million dollars in 2010 (+331%). During the technological breakthrough period from 2011 to 2017, the average annual growth rate was 34.6%, peaking at 853 million dollars in 2017. From 2018 to 2023, during the clinical translation phase, the funding amounts stabilized in the range of 470 to 790 million dollars, reflecting a sustained shift in strategic focus towards therapeutic development. The influence and recognition of scientific literature will accumulate over time and eventually stabilize. As shown in figure 2B, the annual publication trend curve for PD-related mitophagy research is flattening. However, these predictions should be treated with caution. Although the nonlinear fitting curve provides robust predictions based on historical data, the dynamic and complex nature of scientific research cannot be overlooked. Actual trends may be influenced by a variety of internal and external factors. External factors include changes in research funding, policy adjustments, or global events, all of which can impact the scientific publication process. Internal factors involve technological advancements in related fields, the evolution of research methods, or shifts in the academic community's research focus.

**Global collaboration and regional contributions**

The analysis of global research collaboration on mitophagy in PD reveals a dynamic interplay of productivity, influence, and regional specialization.

The United States serves as the central hub of global Parkinson's research collaboration, reflected in its leading publication volume (479), betweenness centrality (0.58), and total link strength (312). Seminal work established the PINK1-Parkin axis: Narendra et al.[36] described PINK1 stabilization on depolarized mitochondria as a critical trigger for Parkin recruitment, complemented by Matsuda[19] showing that PINK1-dependent mitochondrial localization releases Parkin's latent ubiquitin ligase activity. Youle and Narendra[22] synthesized these mechanisms into an evolutionary conserved framework for mitochondrial quality control. Subsequent investigations expanded this paradigm: Chen[45] identified mitofusin 2 (Mfn2) as a PINK1-phosphorylated Parkin receptor, Zhang et al.[59] revealed BNIP3's role in stabilizing full-length PINK1 to facilitate Parkin recruitment, and Harbauer et al.[60] uncovered neuron-specific PINK1 mRNA trafficking via SYNJ2/SYNJ2BP for local mitophagy activation. Regulatory mechanisms were further elucidated by Bingol et al.[61], who characterized USP30 as a deubiquitinase antagonizing Parkin-mediated ubiquitylation, Heo et al.[62] detailing the TBK1-dependent amplification cascade via OPTN/NDP52 recruitment, and Fiesel et al. [63] demonstrating that the PINK1-G411A substitution enhances ubiquitin phosphorylation kinetics and mitophagy efficiency through optimized substrate receptivity. Pathophysiological connections were robustly characterized: Hsieh et al.[64] linked impaired Miro degradation to defective mitophagy in familial and sporadic PD using iPSC-derived neurons, while Grassi et al.[65] identified neurotoxic Pα-syn* aggregates inducing mitochondrial damage and fragmentation that trigger Parkin-dependent mitophagy. These mechanistic insights directly informed therapeutic development: Fang et al. [66] validated that USP30 knockout or pharmacologic inhibition protects dopaminergic neurons in α-synucleinopathy models. While China ranks second in productivity (353 publications), its relatively low betweenness centrality (0.13) and total link strength (102) suggest a focus on domestic or regionally clustered research, indicating a need for deeper integration into global networks. The citation bursts observed in China (2022–2024, strength=20.96) and India (2022–2024, strength=14.3) signal shifting dynamics (figure 3C). China’s surge aligns with its strategic investments in neurodegenerative research. In recent years, Chinese scholars have made significant contributions to elucidating the molecular mechanisms and therapeutic strategies targeting mitophagy in PD. In the context of core regulatory pathways, Wang et al. revealed that PTEN-L acts as a novel phosphatase to inhibit PINK1-Parkin-mediated mitophagy through ubiquitin dephosphorylation[67]. Complementary studies by Huang et al. and Niu et al. further demonstrated the critical roles of metabolic enzymes (PANK2)[68] and deubiquitinating regulators (USP33)[69] in modulating PINK1-Parkin signaling, highlighting the dynamic interplay between ubiquitination and mitochondrial quality control. Regarding mitochondrial dynamics, the Chen team systematically established the involvement of Drp1-mediated fission in paraquat-induced neuronal damage[70,71], while Han et al. identified PINK1-dependent phosphorylation of Drp1 at Ser616 as a key modulator of mitochondrial morphology[72]. In therapeutic development, Liu et al. reported that lovastatin enhances SHP2-mediated mitophagy to alleviate parkinsonism in murine models[73]. Innovative nanotechnology-driven approaches, such as sequence-targeted lycopene nanodots[74] and single-atom nanocatalytic platforms[75], were designed to promote pro-survival mitophagy and suppress neuroinflammation, respectively. Epigenetic studies uncovered non-coding RNA networks, including the circEPS15/miR-24-3p axis[76] and LncRNA NR_030777[71], which regulate mitophagy through ATG12 and CDK1 pathways. Notably, Bao et al. proposed a non-canonical mitochondrial quality control mechanism involving mitolysosome exocytosis[77], whereas Zhang et al. and Han et al. elucidated crosstalk between mitophagy and ferroptosis via NKAα1 inhibition[78] and Nrf2-mediated lipid peroxidation regulation[79]. These findings collectively provide a multifaceted framework for understanding PD pathogenesis and advancing mitochondrion-targeted therapies.

India’s growth, though starting from a smaller base, reflects rising interest in PD epidemiology and cost-effective therapeutic strategies, such as repurposed mitochondrial enhancers. Annual publication trends (figure 3D) further highlight this geographic diversification: the U.S. maintains steady growth, China exhibits exponential output since 2018, and India shows accelerating contributions post-2020.Recent years have witnessed significant progress from Indian researchers in understanding mitophagy regulation and therapeutic applications in PD. By establishing drosophila models, studies revealed that Rab11 modulates mitochondrial quality control through the Parkin/PINK1 signaling pathway[80]. Rodent studies demonstrated that pharmacological inhibition of deubiquitinating enzyme USP14 markedly amplified mitophagic activity and alleviated dopaminergic neurodegeneration[81]. Furthermore, SH-SY5Y cell-based investigations elucidated that andrographolide suppresses NLRP3 inflammasome activation via Parkin-mediated mitophagy[82]. These findings collectively unravel the complexity of mitophagic networks, providing experimental foundations for developing targeted nanodelivery systems and epigenome-modulating strategies.

In Europe, England (170 publications) and Germany (122 publications), demonstrate strong collaboration (total link strengths of 188 and 153, respectively). However, their relatively lower centrality values compared to USA highlight a gap in facilitating cross-regional knowledge exchange. England’s key studies include. Ziviani et al.[83] demonstrate conserved PINK1-dependent mitofusin ubiquitination in Drosophila models. Lee et al.[84] challenge prevailing paradigms by documenting persistent basal mitophagy in Pink1/parkin-null organisms, despite progressive PD pathology. Usher et al.[85] further validate non-canonical phospho-ubiquitin turnover independent of autophagy machinery. Essential regulatory components are further defined through Burchell et al.[86] who demonstrate Fbxo7-Parkin interactions critical for mitophagy initiation in PARK15-linked PD, while Martinez et al. [87] identify CISD1 inhibition as a therapeutic strategy to rescue PD-associated mitophagy failure. German research has systematically delineated the regulatory network and pathological mechanisms of the PINK1/Parkin pathway in PD. The Geisler group demonstrated that PD-associated PINK1/Parkin mutations impair mitophagy, leading to accumulation of damaged mitochondria[88]. Meissner et al. identified the mitochondrial protease PARL as a negative regulator that cleaves PINK1[89], while the Burbulla team revealed that mortalin deficiency triggers mitochondrial proteotoxic stress and activates the unfolded protein response (UPR(mt)), which can be rescued by Parkin/PINK1 overexpression[90]. Mueller-Rischart reported a non-mitophagic function of Parkin, showing it maintains mitochondrial integrity via linear ubiquitination of NEMO to activate NF-κB signaling[91]. Regarding disease progression, Borsche’s cohort study established that PRKN/PINK1 mutations induce mtDNA release and cGAS-STING-mediated inflammation, validating serum IL-6 and circulating cell-free mtDNA as diagnostic and progression biomarkers[92], whereas Torres-odio demonstrated in mouse models that PINK1 deficiency drives temporal pathology cascades—from early spliceosome dysfunction and mid-stage ubiquitin-proteasome dysregulation to late neuroinflammation [93]. For therapeutic targeting, Polachova developed PARL-specific ketoamide inhibitors to activate PINK1/Parkin-dependent mitophagy[94], and Roverato proposed inhibiting the inflammation-induced FAT10-Parkin axis to restore mitochondrial clearance[95].

**Institutional impact and collaborative strategies**

From the perspective of institutional distribution, research on mitophagy in PD exhibits a multipolar pattern, yet with significant geographical concentration. Institutions from North America and Europe dominate the field, with four US institutions ranking in the top ten (National Institute of Neurological Disorders and Stroke[NINDS], University of Pittsburgh, Mayo Clinic, and Johns Hopkins University). Collectively, these institutions account for 51.2% of the total citations among the top ten, with NINDS alone amassing 10,427 citations, thereby underscoring its academic leadership. Among European institutions, University College London (UCL) stands out as a core hub in the global collaborative network, with a total link strength of 128. Its extensive collaborations likely stem from the integration of clinical resources and fundamental research capabilities.

As depicted in the institutional collaboration network (figure 4A), institutions such as University College London (total link strength of 128) and the Mayo Clinic (total link strength of 99) occupy core hub positions. Their extensive collaborative ties indicate that the deep integration of clinical and basic research is pivotal in driving the translation of mitophagy research into therapeutic strategies. However, the collaboration network remains predominantly intra-national clusters (e.g.,US and German institutions forming distinct clusters), suggesting that geographical proximity and shared research funding systems may still be the primary drivers of collaboration, despite the global demand for PD research. The increased activity of Chinese institutions in recent years is evidenced by the concentrated distribution of red nodes (representing recent publications) in figure 4B. The Chinese Academy of Sciences (CAS, 29 publications) stands as a representative of China's research productivity, ranking sixth globally in terms of publication output. It should be noted that the literature inclusion threshold of VOSviewer (≥5 publications) may underestimate the collaborative potential of emerging institutions,as there may be initial collaborations in the actual research network that are not visualized.

For researchers newly entering the field of mitophagy in PD, priority should be given to core institutions with sustained high productivity. For instance, McGill University (with 52 publications) and University College London (with 45 publications) not only maintain stable research output but also their high total link strength (84 and 128, respectively) indicates that their achievements are mostly generated in a collaborative innovation environment. Particular attention should be paid to the 27 publications from the National Institute of Neurological Disorders and Stroke (NINDS), with an average citation per paper reaching 386 times, in order to comprehend the core theoretical framework of the field. Based on the bibliometric analysis results, a multi-dimensional strategy should be adopted when selecting collaborative teams. First, high-link-strength hub institutions should be identified, with priority given to teams with a total link strength greater than 80 and an average annual publication output exceeding five papers (such as University College London and the University of Tübingen). These institutions are characterized by dense node connection lines and spanning multiple research clusters in the collaboration network depicted in figure 4A. Second, highly active institutions should be tracked, with a focus on red nodes in figure 4B that have an average publication year after 2018, such as the Chinese Academy of Sciences, which has shown a significant fluctuating upward trend in publication output between 2018 and 2022. Finally, the compatibility of international cooperation should be assessed, with teams that have at least five co-authored papers with international partners being selected. It is recommended that new researchers prioritize teams with a total link strength higher than 50 and a record of transnational co-authorship in the past three years when choosing institutions, in order to enhance the visibility and translational efficiency of research outcomes. By integrating the three dimensions of institutional influence, technical complementarity, and cooperation maturity, researchers can systematically optimize their team selection decisions.

**Journal distribution and research synergies**

The journal analysis shows that mitophagy research in PD is highly concentrated, with 22 core journals (Zone 1) accounting for most publications. *Autophagy* (IF=14.3,Q1) stands out with 55 publications, establishing its authority in mitophagy research. Figure 7A maps the core structure of PD mitophagy research. The red, blue, and green clusters are evenly distributed, signifying their equal importance. The red cluster represents molecular neurobiology, the blue cluster focuses on autophagy mechanisms, and the green cluster pertains to PD genetics and pathology. In contrast, the smaller yellow cluster, located between the red and blue clusters, emphasizes PD translational research, serving as a bridge that connects fundamental mechanisms with disease pathology. The yellow nodes, which symbolize elements of translational research, are interspersed throughout the main clusters, underscoring the robust connections between translational research and other critical areas of study.

Notably, top-tier interdisciplinary journals like *Nature*, despite their relatively low volume of publications (figure 7B), often feature breakthrough studies, as indicated by their high average normalized citation rates (deep red nodes). *Nature*'s research on mitophagy in PD has been pivotal in integrating molecular mechanisms, pathological associations, and targeted therapies. Seminal studies include the elucidation of the PARKIN-dependent ubiquitylome in response to mitochondrial depolarization[96], genome-wide RNAi screens identifying regulators of parkin upstream of mitophagy[97], and the role of USP30 in opposing parkin-mediated mitophagy[61]^.^ Structural studies have detailed the activation mechanism of Parkin by phosphorylated ubiquitin[98], the interaction mode of PINK1 with ubiquitin[99], and the mechanism of Parkin activation by PINK1[100]. These studies have precisely explained the mechanisms of early-onset PD mutations. Research has also expanded into the gut-brain axis, revealing how intestinal infections can trigger neuronal immune attacks via mitochondrial antigens[101]. Cryo-electron microscopy capturing the full activation pathway of PINK1[102] provides an atomic-level blueprint for targeted interventions. In contrast, high-volume journals like *Autophagy* focus more on in-depth analysis of mechanistic details, forming a complementary pattern where top-tier journals set the direction and specialized journals deepen the research.

**Author productivity and collaborative networks**

As shown in figure 9, the actual distribution of author productivity significantly deviates from Lotka's Law, with a much higher proportion of authors contributing only one publication (78.6% vs. the predicted 62%). This suggests a large number of short-term participants or interdisciplinary collaborators. This pattern may result from the need to integrate knowledge across multiple disciplines, such as neurodegenerative disease mechanisms, cell biology, and molecular genetics. Additionally, the high average number of co-authors per publication (6.9) suggests a strong collaborative structure, leading to more one-time contributors. Among authors with two or more publications, the actual proportions are consistently lower than Lotka's Law predictions, indicating a highly concentrated core research group in the field.

The research area is marked by a collaborative network centered on key scholars and institutions. Hattori N from Juntendo University and Youle RJ from the National Institute of Neurological Disorders and Stroke (NINDS) have emerged as cornerstones of the field. Hattori N has published 28 papers, which account for a significant proportion of Juntendo University’s total publications in this area (totaling 34 papers). His extensive internal collaborations within the university have played a crucial role in shaping the institution’s overall contributions to the field. Meanwhile, Youle RJ's work has been highly influential, with a citation count of 12,235. Hattori N's team focuses on the roles of PINK1 and Parkin genes in PD and their functions in mitochondrial quality control. Their studies have revealed that PINK1 phosphorylates the ubiquitin-like domain (Ubl) of Parkin upon loss of mitochondrial membrane potential, promoting Parkin translocation to and activation on mitochondria, thereby triggering mitophagy[103]. They also found that mitochondrial dysfunction caused by PINK1 deficiency is associated with defects in the respiratory chain rather than proton leak[104]. In animal models, Hattori N further confirmed that Parkin deficiency impairs mitochondrial turnover and leads to dopaminergic neuronal loss[105]. Youle RJ's team has elucidated the critical mechanisms of PINK1 and Parkin in regulating mitophagy[36]. They discovered that PINK1 stability is regulated by mitochondrial membrane potential and identified several proteins regulating the PINK1/Parkin pathway, such as TOMM7, HSPA1L, and BAG4[97]. They also found that Rab protein cycling in the endoplasmic reticulum plays an important role in Parkin-mediated mitophagy[106]. These findings have advanced our understanding of PD pathogenesis and laid a solid foundation for the field. Notably, the institutional distribution shows that McGill University (with Fon EA and Trempe JF) and Mayo Clinic (with Springer W and Fiesel FC) each have two scholars in the top ten. Their total link strengths (184/120 for McGill University and 273/256 for Mayo Clinic) are relatively high, indicating stable and efficient collaborative mechanisms within these institutions.

The co-authorship network map (figure 10A) shows a hierarchical structure with scholars like Hattori N, Youle RJ, Springer W, and Fon EA as central hubs. These researchers have published at least 20 papers each and connect multiple clusters, acting as bridges in cross-team collaborations. Emerging peripheral networks represent potential research frontiers, such as new genetic regulators or therapeutic targets. These groups, though currently small, may play a pivotal role in future research and require sustained funding and mentorship to enhance their impact. Figure 11B highlights Youle RJ's dominance through a color gradient of average normalized citations (darker red indicates higher impact). Node colors also reflect differences in research timelines: Fon EA and Trempe JF have redder nodes (more recent publications), while Youle RJ and Springer W have bluer nodes (earlier publications).

Collaboration and institutional support are key to advancing research in this field. While top scholars have made significant contributions, emerging researchers and small teams need support to prevent knowledge gaps. Interdisciplinary integration can improve research efficiency and address the complexity of PD pathogenesis.

**Hotspots and frontiers**

**Core mechanisms and dominant pathways**

Keyword analysis in the field of mitophagy in PD reveals dynamic shifts in research paradigms and core scientific questions. Research has consistently focused on the PINK1-Parkin pathway, as evidenced by the high frequency and strong co-occurrence of keywords "PINK1" (494 occurrences) and "Parkin" (357 occurrences). This indicates that the pathway has continuously dominated the research framework since its early discovery and highlights the complexity of its regulatory mechanisms. PINK1 is a mitochondrial protein kinase that accumulates on the outer mitochondrial membrane when mitochondria are damaged or depolarized, recruiting Parkin. Parkin then ubiquitinates damaged mitochondria, marking them for autophagic degradation[48,50,96].This pathway is not only involved in mitochondrial quality control but also plays roles in oxidative stress and metabolic regulation[107,108]. The PINK1-Parkin pathway also interacts with other autophagy pathways, such as those mediated by FUNDC1 and BNIP3L, to maintain cellular metabolic balance[109,110,111]. In PD, loss of PINK1 and Parkin function leads to impaired mitophagy, resulting in neuronal damage and death[112].Therapeutic strategies targeting the PINK1-Parkin pathway are emerging as a research focus. Activation of this pathway can enhance mitophagy, improving mitochondrial function and cell survival. For example, certain small molecules have been identified to activate the PINK1-Parkin pathway, promoting mitochondrial clearance and cellular protection[113,114,115]. Inhibition of the deubiquitinase USP30 has been shown to enhance Parkin activity, restoring mitochondrial quality[116,117,118].

**Mitochondrial quality control**

The prominence of keywords such as "mitochondria" (rank 5), "mitochondrial dysfunction" (rank 11), and "damaged mitochondria" (rank 18) reflects a paradigm shift in research from a singular focus on autophagy mechanisms to an integrated multimodal quality control network. Mitochondrial quality control (MQC) integrates mitochondrial dynamics (fusion and fission), mitochondrial biogenesis, and mitophagy to maintain mitochondrial integrity and cellular homeostasis[119].

Mitochondrial fission, mediated by Drp1, is a critical step in the autophagy process, facilitating the clearance of damaged mitochondria to maintain cellular health[120]. Studies show that fission and autophagy are closely linked: fission not only provides sufficient mitochondrial fragments for autophagy but also regulates autophagy efficiency through signaling pathways[121]. Inhibition of mitochondrial fission impairs autophagy, exacerbating cellular damage and death[122]. Conversely, mitochondrial fusion, mediated by MFN1/2, delays mitophagy initiation through content mixing, creating a "damage buffering" mechanism. By diluting abnormal signals (e.g.,oxidatively damaged proteins), fusion postpones autophagy[123,124]. However, MFN2 has been shown to have dual functions in cardiomyocyte injury: it maintains mitochondrial quality through fusion and induces mitophagy by activating Parkin translocation and phosphorylation, clearing damaged mitochondria and protecting cells[125]. Recent studies have revealed that the imbalance between mitophagy and mitochondrial biogenesis is a core mechanism underlying dopaminergic neuronal degeneration. PINK1/Parkin mutations not only impair mitophagy but also inhibit PGC-1α activity through PARIS protein accumulation[126,127].This dual defect creates a vicious cycle: impaired mitophagy leads to the accumulation of damaged mitochondria, while insufficient biogenesis prevents neurons from compensating with functional mitochondria, ultimately exacerbating oxidative stress and energy metabolic collapse.

**α-synuclein**

PD is marked by abnormal α-synuclein aggregation, which is key to its pathogenesis. Lurette et al. controlled α-synuclein aggregation using optogenetic tools and found it significantly impacts mitophagy. The aggregates cause mitochondrial depolarization, reduced ATP, fission, and mitophagy via cardiolipin externalization, and lower mitochondrial content in dopaminergic neurons and mouse midbrains. This shows that aggregation, not overexpression, of α-synuclein drives mitophagy and mitochondrial dysfunction, offering new insights into PD[128]. Additionally, the abnormal accumulation of α-synuclein disrupts mitochondrial function and disturbs the dynamic balance of mitophagy, thereby promoting neuronal degeneration[129,130,131]. Shaltouki et al. analyzed PD patient brains, neurons, and fly models and found that α-synuclein accumulation upregulates Miro protein levels. Miro, a mitochondrial outer membrane protein, is involved in mitochondrial movement and clearance of damaged mitochondria. In PD neurons, Miro abnormally accumulates on mitochondria, delaying mitophagy. α-synuclein interacts with Miro via its N-terminus, driving Miro upregulation. Reducing Miro levels rescues mitophagy and neurodegeneration. This study underscores the role of mitochondrial-associated α-synuclein in PD and identifies Miro as a potential therapeutic target[132]. Abnormal aggregation of α-synuclein activates the p38 MAPK pathway, phosphorylating Parkin at Ser131 and impairing its function. This disrupts mitophagy, worsening mitochondrial dysfunction and neuronal death in the A53T α-synuclein model. Inhibiting p38 MAPK activity reduces apoptosis, restores mitochondrial membrane potential, and increases synaptic density[133]. Yin et al. showed that Nur77 is key to regulating α-synuclein aggregation and mitophagy using STI571 and antibodies. STI571 inhibits PHB2 Y121 phosphorylation, reduces α-synuclein aggregates, and boosts autophagy. Nur77 moves to mitochondria in the presence of α-synuclein, enhancing PHB-mediated mitophagy and reducing mitochondrial dysfunction. In α-synuclein PFF mouse models, Nur77 overexpression lowers pS129-α-synuclein levels and protects dopaminergic neurons, likely via the p-c-Abl/p-PHB2 Y121 axis. This suggests Nur77 and STI571 could be potential therapeutic targets for PD[134].

The natural compound quercetin upregulates PINK1/Parkin expression, reduces α-synuclein aggregation, and improves mitochondrial quality control in 6-OHDA-induced models[135]. These findings suggest that modulating the balance between mitophagy and α-synuclein aggregation may represent a novel therapeutic strategy for PD.

**Neuroinflammation and ferroptosis**

The rising trends of "neuroinflammation" and "ferroptosis" (Figure 12 and 13A) highlight the growing emphasis on the interplay between multiple mechanisms in the field. Mitophagy, primarily mediated by the PINK1/Parkin pathway, is crucial for maintaining mitochondrial homeostasis. Dysfunction in this pathway leads to the accumulation of damaged mitochondria, resulting in mitochondrial DNA (mtDNA) leakage and reactive oxygen species (ROS) accumulation. These changes activate the cGAS-STING signaling pathway and the NLRP3 inflammasome, promoting the release of pro-inflammatory cytokines such as IL-1β and IL-6, and inducing microglial polarization towards the pro-inflammatory M1 phenotype[136,137,138,139]. Targeting mitophagy has been shown to effectively alleviate neuroinflammation. For instance, the natural compound Urolithin A enhances PINK1/Parkin-dependent mitophagy and inhibits NLRP3 inflammasome activation[138]. Similarly, Repaglinide activates mitophagy and modulates endoplasmic reticulum stress, inhibiting glial cell activation and neuroinflammation, thereby reducing dopaminergic neuronal apoptosis[140].

Ferroptosis, a form of iron-dependent lipid peroxidation-driven cell death, is closely intertwined with mitophagy in neurodegenerative processes. ROS accumulation from mitochondrial dysfunction can exacerbate neuronal damage via ferroptosis pathways. For example, neurotoxins like rotenone induce excessive ROS generation, activating ferroptosis markers (e.g.,GPX4 downregulation, COX2 and NCOA4 upregulation) while inhibiting autophagy flux and enhancing mitophagy markers (e.g.,LC3 and p62), ultimately leading to dopaminergic neuronal death[141]. Iron metabolism disturbances, such as iron deposition and transferrin receptor abnormalities, form a vicious cycle with mitophagy dysregulation in disease models. For instance, bifenthrin exposure exacerbates the synergistic effects of mitophagy and ferroptosis by binding to iron transport proteins (Tf) and GPX4[142]. Additionally, ferritin heavy chain 1 (FTH1) regulation is prominent in 6-OHDA models, where inhibiting ferritinophagy reduces ferroptosis and improves mitochondrial function, suggesting therapeutic potential in targeting iron metabolism and mitochondrial quality control[143]. These findings indicate that mitophagy and ferroptosis are not isolated events in neurodegenerative pathology but form a positive feedback loop through mechanisms involving oxidative stress, iron homeostasis imbalance, and energy metabolism disruption. Future research should focus on elucidating the spatiotemporal regulatory networks between these processes to inform the development of dual-target therapeutic strategies.

**Challenges and future directions**

Despite the prominence of keywords such as "ubiquitin" (rank 13) and "oxidative stress" (rank 6), which point to numerous potential therapeutic targets, the frequency of terms related to translational medicine, such as "therapeutic targets" and "biomarkers" does not match their scientific importance. This reflects systemic barriers in translating preclinical findings into clinical applications. The mechanisms underlying mitophagy are complex and dynamically balanced, making precise modulation challenging. For instance, although the PINK1/Parkin pathway is extensively studied, most PD patients lack mutations in these genes[144]. Additionally, since PD pathology involves the interplay of multiple pathways (such as α-synuclein aggregation and oxidative stress), targeting mitophagy alone may be insufficient to halt disease progression. Synergistic regulation of other mechanisms (such as mitochondrial biogenesis or anti-inflammatory pathways) is needed, but the complexity and risk of side effects associated with multi-target drug development are significantly increased. Despite the challenges associated with clinical development, small-molecule approaches aimed at selectively enhancing mitophagy, such as USP30 inhibitors and PINK1 activators, are now entering phase I clinical trials[145].

# **Limitations**

# Our study provides a comprehensive overview of mitophagy research in PD using bibliometric techniques. However, several limitations are inherent to our approach. Firstly, our dataset is derived solely from the Web of Science Core Collection, potentially omitting relevant articles from other databases. Secondly, our analysis is confined to English-language literature, which may introduce bias[146]. Lastly, the presence of homonymous authors or different expressions of the same author may affect the accuracy of our collaborative network analysis.

# **Conclusion**

Our analysis of mitophagy research in PD from 2007 to 2024 reveals several key findings. The United States is the leader in terms of publication output, followed by China. McGill University is the most prolific institution, while the journal *Autophagy* is the most frequent publication venue, with *International Journal of Molecular Sciences* ranking second. Hattori Nobutaka, is the most prolific author, followed by Edward A. Fon. Research foci include "pink1/parkin", "mitochondrial quality control" and α-synuclein, with neuroinflammation and ferroptosis emerging as hotspots. These findings provide a comprehensive overview of the field, highlighting critical insights into current research trajectories. We anticipate that these insights will help researchers better understand prevailing trends in mitophagy research in PD and guide future investigative endeavors.

**Acknowledgements** This work was supported by the Shanghai Municipal Commission of Health (shzyyzdxk-2024108).

**Author Contribution** J.Q.Z. conceptualized the study, designed the research framework, conducted the literature search and data collection, analyzed the data, interpreted the results, and wrote the initial draft. Q.W., Y.C. and H.M.S.,assisted in data analysis and interpretation, and edited the manuscript. S.F.X. supervised the study, conducted the final data analysis and interpretation, wrote and edited the manuscript, and managed the submission process.

**Funding** This work was supported by the Shanghai Municipal Commission of Health (shzyyzdxk-2024108).

**Data Availability** No datasets were generated or analysed during the current study.

**Declarations**

**Conflict of Interest** The authors declare no competing interests.

**References**

1. Luo Y, Qiao L, Li M, Wen X, Zhang W, Li X (2025) Global, regional, national epidemiology and trends of Parkinson’s disease from 1990 to 2021: findings from the Global Burden of Disease Study 2021. Front Aging Neurosci 16:1498756. <https://doi.org/10.3389/fnagi.2024.1498756>
2. Yang W, Hamilton JL, Kopil C, Beck JC, Tanner CM, Albin RL, Ray Dorsey E, Dahodwala N, Cintina I, Hogan P, Thompson T (2020) Current and projected future economic burden of Parkinson’s disease in the U.S. NPJ Parkinson’s Dis 6:15. <https://doi.org/10.1038/s41531-020-0117-1>
3. Li G, Ma J, Cui S, He Y, Xiao Q, Liu J, Chen S (2019) Parkinson’s disease in China: a forty-year growing track of bedside work. Transl Neurodegener 8:22. <https://doi.org/10.1186/s40035-019-0162-z>
4. Steiner JA, Angot E, Brundin P (2011) A deadly spread: cellular mechanisms of α -synuclein transfer. Cell Death Differ 18(9):1425–1433. <https://doi.org/10.1038/cdd.2011.53>
5. Dehay B, Bourdenx M, Gorry P, Przedborski S, Vila M, Hunot S, Singleton A, Olanow CW, Merchant KM, Bezard E, Petsko GA, Meissner WG (2015) Targeting α -synuclein for treatment of Parkinson’s disease: mechanistic and therapeutic considerations. Lancet Neurol 14(8):855–866. <https://doi.org/10.1016/S1474-4422(15)00006-X>
6. Perier, C., & Vila, M. (2012). Mitochondrial biology and Parkinson's disease. Cold Spring Harbor perspectives in medicine, 2(2), a009332. <https://doi.org/10.1101/cshperspect.a009332>
7. Langeh, U., Kumar, V., Kumar, A., Kumar, P., Singh, C., & Singh, A. (2022). Cellular and Mitochondrial Quality Control Mechanisms in Maintaining Homeostasis in Aging. Rejuvenation research, 25(5), 208–222. https://doi.org/10.1089/rej.2022.0027
8. Schapira AH, Cooper JM, Dexter D, Jenner P, Clark JB, Marsden CD (1989) Mitochondrial complex I deficiency in Parkinson’s disease. Lancet 1(8649):1269. <https://doi.org/10.1016/s0140-6736(89)92366-0>
9. Valente EM, Abou-Sleiman PM, Caputo V, Muqit MM, Harvey K, Gispert S, Ali Z, Del Turco D, Bentivoglio AR, Healy DG, Albanese A, Nussbaum R, González-Maldonado R, Deller T, Salvi S, Cortelli P, Gilks WP, Latchman DS, Harvey RJ, Dallapiccola B, Wood NW (2004) Hereditary early-onset Parkinson’s disease caused by mutations in PINK1. Science 304(5674):1158–1160. <https://doi.org/10.1126/science.1096284>
10. Kitada T, Asakawa S, Hattori N, Matsumine H, Yamamura Y, Minoshima S, Yokochi M, Mizuno Y, Shimizu N (1998) Mutations in the parkin gene cause autosomal recessive juvenile parkinsonism. Nature 392(6676):605–608. <https://doi.org/10.1038/33416>
11. Ibáñez, P., Lesage, S., Lohmann, E., Thobois, S., De Michele, G., Borg, M., Agid, Y., Dürr, A., Brice, A., & French Parkinson's Disease Genetics Study Group (2006). Mutational analysis of the PINK1 gene in early-onset parkinsonism in Europe and North Africa. Brain : a journal of neurology, 129(Pt 3), 686–694. <https://doi.org/10.1093/brain/awl005>
12. Gelmetti, V., Ferraris, A., Brusa, L., Romano, F., Lombardi, F., Barzaghi, C., Stanzione, P., Garavaglia, B., Dallapiccola, B., & Valente, E. M. (2008). Late onset sporadic Parkinson's disease caused by PINK1 mutations: clinical and functional study. Movement disorders : official journal of the Movement Disorder Society, 23(6), 881–885. <https://doi.org/10.1002/mds.21960>
13. Kasten, M., Hartmann, C., Hampf, J., Schaake, S., Westenberger, A., Vollstedt, E. J., Balck, A., Domingo, A., Vulinovic, F., Dulovic, M., Zorn, I., Madoev, H., Zehnle, H., Lembeck, C. M., Schawe, L., Reginold, J., Huang, J., König, I. R., Bertram, L., Marras, C., … Klein, C. (2018). Genotype-Phenotype Relations for the Parkinson's Disease Genes Parkin, PINK1, DJ1: MDSGene Systematic Review. Movement disorders : official journal of the Movement Disorder Society, 33(5), 730–741. <https://doi.org/10.1002/mds.27352>
14. Han, R., Liu, Y., Li, S., Li, X. J., & Yang, W. (2023). PINK1-PRKN mediated mitophagy: differences between in vitro and in vivo models. Autophagy, 19(5), 1396–1405. https://doi.org/10.1080/15548627.2022.2139080
15. Dauer, W., & Przedborski, S. (2003). Parkinson's disease: mechanisms and models. Neuron, 39(6), 889–909. <https://doi.org/10.1016/s0896-6273(03)00568-3>
16. Shimoji, M., Zhang, L., Mandir, A. S., Dawson, V. L., & Dawson, T. M. (2005). Absence of inclusion body formation in the MPTP mouse model of Parkinson's disease. Brain research. Molecular brain research, 134(1), 103–108. https://doi.org/10.1016/j.molbrainres.2005.01.012
17. Dawson TM, Dawson VL (2017) Mitochondrial Mechanisms of Neuronal Cell Death: Potential Therapeutics. Annu Rev Pharmacol Toxicol 57:437–454. <https://doi.org/10.1146/annurev-pharmtox-010716-105001>
18. Onishi M, Okamoto K (2021) Mitochondrial clearance: mechanisms and roles in cellular fitness. FEBS Lett 595(8):1239–1263. <https://doi.org/10.1002/1873-3468.14060>
19. Matsuda N, Sato S, Shiba K, et al. PINK1 stabilized by mitochondrial depolarization recruits Parkin to damaged mitochondria and activates latent Parkin for mitophagy. J Cell Biol. 2010;189(2):211-21. doi:10.1083/jcb.200910140 <https://pubmed.ncbi.nlm.nih.gov/20404107>
20. Lechado-Terradas, A., Schepers, S., Zittlau, K. I., Sharma, K., Ok, O., Fitzgerald, J. C., Geimer, S., Westermann, B., Macek, B., & Kahle, P. J. (2022). Parkin-dependent mitophagy occurs via proteasome-dependent steps sequentially targeting separate mitochondrial sub-compartments for autophagy. Autophagy reports, 1(1), 576–602. <https://doi.org/10.1080/27694127.2022.2143214>
21. Miyashita, H., Oikawa, D., Terawaki, S., Kabata, D., Shintani, A., & Tokunaga, F. (2021). Crosstalk Between NDP52 and LUBAC in Innate Immune Responses, Cell Death, and Xenophagy. Frontiers in immunology, 12, 635475. <https://doi.org/10.3389/fimmu.2021.635475>
22. Youle, R. J., & Narendra, D. P. (2011). Mechanisms of mitophagy. Nature reviews. Molecular cell biology, 12(1), 9–14. <https://doi.org/10.1038/nrm3028>
23. Terešak, P., Lapao, A., Subic, N., Boya, P., Elazar, Z., & Simonsen, A. (2022). Regulation of PRKN-independent mitophagy. Autophagy, 18(1), 24–39. <https://doi.org/10.1080/15548627.2021.1888244>
24. Chu, C. T., Ji, J., Dagda, R. K., Jiang, J. F., Tyurina, Y. Y., Kapralov, A. A., Tyurin, V. A., Yanamala, N., Shrivastava, I. H., Mohammadyani, D., Wang, K. Z. Q., Zhu, J., Klein-Seetharaman, J., Balasubramanian, K., Amoscato, A. A., Borisenko, G., Huang, Z., Gusdon, A. M., Cheikhi, A., Steer, E. K., … Kagan, V. E. (2013). Cardiolipin externalization to the outer mitochondrial membrane acts as an elimination signal for mitophagy in neuronal cells. Nature cell biology, 15(10), 1197–1205. <https://doi.org/10.1038/ncb2837>
25. Picca, A., Guerra, F., Calvani, R., Coelho-Júnior, H. J., Landi, F., Bucci, C., & Marzetti, E. (2023). Mitochondrial-Derived Vesicles: The Good, the Bad, and the Ugly. International journal of molecular sciences, 24(18), 13835. https://doi.org/10.3390/ijms241813835
26. Dabravolski SA, Nikiforov NG, Zhuravlev AD, Orekhov NA, Grechko AV, Orekhov AN (2022) Role of the mtDNA Mutations and Mitophagy in Inflammaging. Int J Mol Sci 23(3):1323. <https://doi.org/10.3390/ijms23031323>
27. Singh F, Prescott AR, Rosewell P, Ball G, Reith AD, Ganley IG (2021) Pharmacological rescue of impaired mitophagy in Parkinson’s disease-related LRRK2 G2019S knock-in mice. eLife 10:e67604. <https://doi.org/10.7554/eLife.67604>
28. Chen CC (2006) CiteSpace II: Detecting and visualizing emerging trends and transient patterns in scientific literature. J Am Soc Inf Sci Tec 57(3):359–377. <https://doi.org/10.1002/asi.20317>
29. van Eck NJ, Waltman L (2009) Software survey: VOSviewer, a computer program for bibliometric mapping. Scientometrics 84(2):523–538. <https://doi.org/10.1007/s11192-009-0146-3>
30. Mrvar A, Batagelj V (2016) Analysis and visualization of large networks with program package Pajek. Complex Adapt Syst Model 4(1). <https://doi.org/10.1186/s40294-016-0017-8>
31. Aria M, Cuccurullo C (2017) bibliometrix: An R-tool for comprehensive science mapping analysis. J Informetr 11(4):959–975. <https://doi.org/10.1016/j.joi.2017.08.007>
32. Bradford S (1976) CLASSIC PAPER: Sources of Information on Specific Subjects. Collect Manag 1(3–4):95–104. <https://doi.org/10.1300/j105v01n03_06>
33. Chen C, Leydesdorff L (2013) Patterns of connections and movements in dual-map overlays: A new method of publication portfolio analysis. J Assoc Inf Sci Tech 65(2):334–351. https://doi.org/10.1002/asi.22968
34. Lotka, Alfred J. “The Frequency Distribution of Scientific Productivity.” Journal of the Washington Academy of Sciences 16, no. 12 (1926): 317–23. http://www.jstor.org/stable/24529203
35. Geisler S, Holmström KM, Skujat D, Fiesel FC, Rothfuss OC, Kahle PJ, Springer W (2010) PINK1/Parkin-mediated mitophagy is dependent on VDAC1 and p62/SQSTM1. Nat Cell Biol 12(2):119–131. https://doi.org/10.1038/ncb2012
36. Narendra DP, Jin SM, Tanaka A, et al. (2010) PINK1 is selectively stabilized on impaired mitochondria to activate Parkin. PLoS Biol 8(1):e1000298. <https://doi.org/10.1371/journal.pbio.1000298>
37. Hou Y, Dan X, Babbar M, et al. (2019) Ageing as a risk factor for neurodegenerative disease. Nat Rev Neurol 15(10):565–581. <https://doi.org/10.1038/s41582-019-0244-7>
38. Pickrell AM, Youle RJ (2015) The roles of PINK1, parkin, and mitochondrial fidelity in Parkinson’s disease. Neuron 85(2):257–273. <https://doi.org/10.1016/j.neuron.2014.12.007>
39. Ashrafi G, Schwarz TL (2012) The pathways of mitophagy for quality control and clearance of mitochondria. Cell Death Differ 20(1):31–42. <https://doi.org/10.1038/cdd.2012.81>
40. Dias V, Junn E, Mouradian MM (2013) The role of oxidative stress in Parkinson’s disease. J Parkinson Dis 3(4):461–491. <https://doi.org/10.3233/JPD-130230>
41. Vives-Bauza C, Zhou C, Huang Y, et al. (2009) PINK1-dependent recruitment of Parkin to mitochondria in mitophagy. Proc Natl Acad Sci USA 107(1):378–383. https://doi.org/10.1073/pnas.0911187107
42. Chen H, Chan DC (2009) Mitochondrial dynamics—fusion, fission, movement, and mitophagy—in neurodegenerative diseases. Hum Mol Genet 18(R2):R169–R176. <https://doi.org/10.1093/hmg/ddp326>
43. Tanaka A, Cleland MM, Xu S, et al. (2010) Proteasome and p97 mediate mitophagy and degradation of mitofusins induced by Parkin. J Cell Biol 191(7):1367–1380. <https://doi.org/10.1083/jcb.201007013>
44. Fang EF, Hou Y, Palikaras K, et al. (2019) Mitophagy inhibits amyloid-β and tau pathology and reverses cognitive deficits in models of Alzheimer’s disease. Nat Neurosci 22(3):401–412. <https://doi.org/10.1038/s41593-018-0332-9>
45. Chen Y, Dorn GW (2013) PINK1-phosphorylated mitofusin 2 is a Parkin receptor for culling damaged mitochondria. Science 340(6131):471–475. <https://doi.org/10.1126/science.1231031>
46. Jin SM, Lazarou M, Wang C, et al. (2010) Mitochondrial membrane potential regulates PINK1 import and proteolytic destabilization by PARL. J Cell Biol 191(5):933–942. <https://doi.org/10.1083/jcb.201008084>
47. Wang X, Winter D, Ashrafi G, et al. (2011) PINK1 and Parkin target Miro for phosphorylation and degradation to arrest mitochondrial motility. Cell 147(4):893–906. <https://doi.org/10.1016/j.cell.2011.10.018>
48. Lazarou M, Sliter DA, Kane LA, et al. (2015) The ubiquitin kinase PINK1 recruits autophagy receptors to induce mitophagy. Nature 524(7565):309–314. <https://doi.org/10.1038/nature14893>
49. Koyano F, Okatsu K, Kosako H, et al. (2014) Ubiquitin is phosphorylated by PINK1 to activate parkin. Nature 510(7503):162–166. <https://doi.org/10.1038/nature13392>
50. Narendra D, Tanaka A, Suen DF, Youle RJ (2008) Parkin is recruited selectively to impaired mitochondria and promotes their autophagy. J Cell Biol 183(5):795–803. <https://doi.org/10.1083/jcb.200809125>
51. Kane LA, Lazarou M, Fogel AI, et al. (2014) PINK1 phosphorylates ubiquitin to activate Parkin E3 ubiquitin ligase activity. J Cell Biol 205(2):143–153. https://doi.org/10.1083/jcb.201402104
52. Clark, I. E., Dodson, M. W., Jiang, C., Cao, J. H., Huh, J. R., Seol, J. H., Yoo, S. J., Hay, B. A., & Guo, M. (2006). Drosophila pink1 is required for mitochondrial function and interacts genetically with parkin. Nature, 441(7097), 1162–1166. <https://doi.org/10.1038/nature04779>
53. Park, J., Lee, S., Lee, S., et al. (2006). Mitochondrial dysfunction in Drosophila PINK1 mutants is complemented by parkin. Nature, 441, 1157–1161. https://doi.org/10.1038/nature04788
54. Poole AC, Thomas RE, Andrews LA, et al. (2008) The PINK1/Parkin pathway regulates mitochondrial morphology. Proc Natl Acad Sci USA 105(5):1638–1643. <https://doi.org/10.1073/pnas.0709336105>
55. Yang, Y., Gehrke, S., Imai, Y., Huang, Z., Ouyang, Y., Wang, J. W., Yang, L., Beal, M. F., Vogel, H., & Lu, B. (2006). Mitochondrial pathology and muscle and dopaminergic neuron degeneration caused by inactivation of Drosophila Pink1 is rescued by Parkin. Proceedings of the National Academy of Sciences of the United States of America, 103(28), 10793–10798. https://doi.org/10.1073/pnas.0602493103
56. Price DJ de Solla (1963) Little Science, Big Science. Columbia University Press, New York Chichester, West Sussex
57. Choi ML, Chappard A, Singh BP, et al. (2022) Pathological structural conversion of α-synuclein at the mitochondria induces neuronal toxicity. Nat Neurosci 25(9):1134–1148. <https://doi.org/10.1038/s41593-022-01140-3>
58. Ma KY, Fokkens MR, Reggiori F, et al. (2021) Parkinson’s disease-associated VPS35 mutant reduces mitochondrial membrane potential and impairs PINK1/Parkin-mediated mitophagy. Transl Neurodegener 10(1):19. <https://doi.org/10.1186/s40035-021-00243-4>
59. Zhang, T., Xue, L., Li, L., Tang, C., Wan, Z., Wang, R., Tan, J., Tan, Y., Han, H., Tian, R., Billiar, T. R., Tao, W. A., & Zhang, Z. (2016). BNIP3 Protein Suppresses PINK1 Kinase Proteolytic Cleavage to Promote Mitophagy. The Journal of biological chemistry, 291(41), 21616–21629. <https://doi.org/10.1074/jbc.M116.733410>
60. Harbauer, A. B., Hees, J. T., Wanderoy, S., Segura, I., Gibbs, W., Cheng, Y., Ordonez, M., Cai, Z., Cartoni, R., Ashrafi, G., Wang, C., Perocchi, F., He, Z., & Schwarz, T. L. (2022). Neuronal mitochondria transport Pink1 mRNA via synaptojanin 2 to support local mitophagy. Neuron, 110(9), 1516–1531.e9. <https://doi.org/10.1016/j.neuron.2022.01.035>
61. Bingol B, Tea JS, Phu L, et al. (2014) The mitochondrial deubiquitinase USP30 opposes parkin-mediated mitophagy. Nature 510(7505):370–375. https://doi.org/10.1038/nature13418
62. Heo, J. M., Ordureau, A., Paulo, J. A., Rinehart, J., & Harper, J. W. (2015). The PINK1-PARKIN Mitochondrial Ubiquitylation Pathway Drives a Program of OPTN/NDP52 Recruitment and TBK1 Activation to Promote Mitophagy. Molecular cell, 60(1), 7–20. <https://doi.org/10.1016/j.molcel.2015.08.016>
63. Fiesel, F. C., Fričová, D., Hayes, C. S., Coban, M. A., Hudec, R., Bredenberg, J. M., Broadway, B. J., Markham, B. N., Yan, T., Boneski, P. K., Fiorino, G., Watzlawik, J. O., Hou, X., McCarty, A. M., Lewis-Tuffin, L. J., Zhong, J., Madden, B. J., Ordureau, A., An, H., Puschmann, A., … Springer, W. (2023). Substitution of PINK1 Gly411 modulates substrate receptivity and turnover. Autophagy, 19(6), 1711–1732. <https://doi.org/10.1080/15548627.2022.2151294>
64. Hsieh, C. H., Shaltouki, A., Gonzalez, A. E., Bettencourt da Cruz, A., Burbulla, L. F., St Lawrence, E., Schüle, B., Krainc, D., Palmer, T. D., & Wang, X. (2016). Functional Impairment in Miro Degradation and Mitophagy Is a Shared Feature in Familial and Sporadic Parkinson's Disease. Cell stem cell, 19(6), 709–724. <https://doi.org/10.1016/j.stem.2016.08.002>
65. Grassi, D., Howard, S., Zhou, M., Diaz-Perez, N., Urban, N. T., Guerrero-Given, D., Kamasawa, N., Volpicelli-Daley, L. A., LoGrasso, P., & Lasmézas, C. I. (2018). Identification of a highly neurotoxic α-synuclein species inducing mitochondrial damage and mitophagy in Parkinson's disease. Proceedings of the National Academy of Sciences of the United States of America, 115(11), E2634–E2643. <https://doi.org/10.1073/pnas.1713849115>
66. Fang, T. Z., Sun, Y., Pearce, A. C., Eleuteri, S., Kemp, M., Luckhurst, C. A., Williams, R., Mills, R., Almond, S., Burzynski, L., Márkus, N. M., Lelliott, C. J., Karp, N. A., Adams, D. J., Jackson, S. P., Zhao, J. F., Ganley, I. G., Thompson, P. W., Balmus, G., & Simon, D. K. (2023). Knockout or inhibition of USP30 protects dopaminergic neurons in a Parkinson's disease mouse model. Nature communications, 14(1), 7295. https://doi.org/10.1038/s41467-023-42876-1
67. Wang L, Cho YL, Tang Y, et al. (2018) PTEN-L is a novel protein phosphatase for ubiquitin dephosphorylation to inhibit PINK1-Parkin-mediated mitophagy. Cell Res 28(8):787–802. <https://doi.org/10.1038/s41422-018-0056-0>
68. Huang Y, Wan Z, Tang Y, et al. (2022) Pantothenate kinase 2 interacts with PINK1 to regulate mitochondrial quality control via acetyl-CoA metabolism. Nat Commun 13(1):2412. <https://doi.org/10.1038/s41467-022-30178-x>
69. Niu K, Fang H, Chen Z, et al. (2019) USP33 deubiquitinates PRKN/parkin and antagonizes its role in mitophagy. Autophagy 16(4):724–734. <https://doi.org/10.1080/15548627.2019.1656957>
70. Chen N, Guo Z, Luo Z, et al. (2020) Drp1-mediated mitochondrial fission contributes to mitophagy in paraquat-induced neuronal cell damage. Environ Pollut 272:116413. <https://doi.org/10.1016/j.envpol.2020.116413>
71. Chen N, Hu H, Tang J, et al. (2024) LncRNA NR_030777 promotes mitophagy by targeting CDK1-related mitochondrial fission and ATG12 to attenuate paraquat-induced Parkinson’s disease. Environ Pollut 349:123875. <https://doi.org/10.1016/j.envpol.2024.123875>
72. Han H, Tan J, Wang R, et al. (2020) PINK1 phosphorylates Drp1S616 to regulate mitophagy-independent mitochondrial dynamics. EMBO Rep 21(8):e48686. <https://doi.org/10.15252/embr.201948686>
73. Liu W, Wang M, Shen L, et al. (2021) SHP2-mediated mitophagy boosted by lovastatin in neuronal cells alleviates parkinsonism in mice. Signal Transduct Tar 6(1):34. <https://doi.org/10.1038/s41392-021-00474-x>
74. Xia X, Li H, Xu X, et al. (2023) Facilitating Pro-survival Mitophagy for Alleviating Parkinson’s Disease via Sequence-Targeted Lycopene Nanodots. ACS Nano 17(18):17979–17995. <https://doi.org/10.1021/acsnano.3c04308>
75. Li B, Bai Y, Yion C, et al. (2023) Single-Atom Nanocatalytic Therapy for Suppression of Neuroinflammation by Inducing Autophagy of Abnormal Mitochondria. ACS Nano 17(8):7511–7529. <https://doi.org/10.1021/acsnano.2c12614>
76. Zhou Y, Liu Y, Kang Z, et al. (2023) CircEPS15, as a sponge of MIR24-3p ameliorates neuronal damage in Parkinson disease through boosting PINK1-PRKN-mediated mitophagy. Autophagy 19(9):2520–2537. <https://doi.org/10.1080/15548627.2023.2196889>
77. Bao F, Zhou L, Zhou R, et al. (2022) Mitolysosome exocytosis, a mitophagy-independent mitochondrial quality control in flunarizine-induced parkinsonism-like symptoms. Sci Adv 8(15):eabk2376. <https://doi.org/10.1126/sciadv.abk2376>
78. Zhang X, Li G, Chen H, et al. (2024) Targeting NKAα1 to treat Parkinson’s disease through inhibition of mitophagy-dependent ferroptosis. Free Radic Biol Med 218:190–204. <https://doi.org/10.1016/j.freeradbiomed.2024.04.002>
79. Han Z, Wang B, Wen YQ, et al. (2024) Acteoside alleviates lipid peroxidation by enhancing Nrf2-mediated mitophagy to inhibit ferroptosis for neuroprotection in Parkinson’s disease. Free Radic Biol Med 223:493–505. <https://doi.org/10.1016/j.freeradbiomed.2024.07.018>
80. Rai P, Roy JK (2022) Rab11 regulates mitophagy signaling pathway of Parkin and Pink1 in the Drosophila model of Parkinson’s disease. Biochem Biophys Res Commun 626:175–186. <https://doi.org/10.1016/j.bbrc.2022.08.027>
81. Banerjee C, Roy M, Mondal R, et al. (2020) USP14 as a Therapeutic Target Against Neurodegeneration: A Rat Brain Perspective. Front Cell Dev Biol 8:727. <https://doi.org/10.3389/fcell.2020.00727>
82. Ahmed S, Kwatra M, Ranjan Panda S, et al. (2020) Andrographolide suppresses NLRP3 inflammasome activation in microglia through induction of parkin-mediated mitophagy in in-vitro and in-vivo models of Parkinson disease. Brain Behav Immun 91:142–158. <https://doi.org/10.1016/j.bbi.2020.09.017>
83. Ziviani, E., Tao, R. N., & Whitworth, A. J. (2010). Drosophila parkin requires PINK1 for mitochondrial translocation and ubiquitinates mitofusin. Proceedings of the National Academy of Sciences of the United States of America, 107(11), 5018–5023. <https://doi.org/10.1073/pnas.0913485107>
84. Lee, J. J., Sanchez-Martinez, A., Martinez Zarate, A., Benincá, C., Mayor, U., Clague, M. J., & Whitworth, A. J. (2018). Basal mitophagy is widespread in Drosophila but minimally affected by loss of Pink1 or parkin. The Journal of cell biology, 217(5), 1613–1622. <https://doi.org/10.1083/jcb.201801044>
85. Usher, J. L., Sanchez-Martinez, A., Terriente-Felix, A., Chen, P. L., Lee, J. J., Chen, C. H., & Whitworth, A. J. (2022). Parkin drives pS65-Ub turnover independently of canonical autophagy in Drosophila. EMBO reports, 23(12), e53552. <https://doi.org/10.15252/embr.202153552>
86. Burchell, V. S., Nelson, D. E., Sanchez-Martinez, A., Delgado-Camprubi, M., Ivatt, R. M., Pogson, J. H., Randle, S. J., Wray, S., Lewis, P. A., Houlden, H., Abramov, A. Y., Hardy, J., Wood, N. W., Whitworth, A. J., Laman, H., & Plun-Favreau, H. (2013). The Parkinson's disease-linked proteins Fbxo7 and Parkin interact to mediate mitophagy. Nature neuroscience, 16(9), 1257–1265. <https://doi.org/10.1038/nn.3489>
87. Martinez, A., Sanchez-Martinez, A., Pickering, J. T., Twyning, M. J., Terriente-Felix, A., Chen, P. L., Chen, C. H., & Whitworth, A. J. (2024). Mitochondrial CISD1/Cisd accumulation blocks mitophagy and genetic or pharmacological inhibition rescues neurodegenerative phenotypes in Pink1/parkin models. Molecular neurodegeneration, 19(1), 12. <https://doi.org/10.1186/s13024-024-00701-3>
88. Geisler, S., Holmström, K. M., Treis, A., Skujat, D., Weber, S. S., Fiesel, F. C., Kahle, P. J., & Springer, W. (2010). The PINK1/Parkin-mediated mitophagy is compromised by PD-associated mutations. Autophagy, 6(7), 871–878. <https://doi.org/10.4161/auto.6.7.13286>
89. Meissner, C., Lorenz, H., Hehn, B., & Lemberg, M. K. (2015). Intramembrane protease PARL defines a negative regulator of PINK1- and PARK2/Parkin-dependent mitophagy. Autophagy, 11(9), 1484–1498. <https://doi.org/10.1080/15548627.2015.1063763>
90. Burbulla, L. F., Fitzgerald, J. C., Stegen, K., Westermeier, J., Thost, A. K., Kato, H., Mokranjac, D., Sauerwald, J., Martins, L. M., Woitalla, D., Rapaport, D., Riess, O., Proikas-Cezanne, T., Rasse, T. M., & Krüger, R. (2014). Mitochondrial proteolytic stress induced by loss of mortalin function is rescued by Parkin and PINK1. Cell death & disease, 5(4), e1180. <https://doi.org/10.1038/cddis.2014.103>
91. Müller-Rischart, A. K., Pilsl, A., Beaudette, P., Patra, M., Hadian, K., Funke, M., Peis, R., Deinlein, A., Schweimer, C., Kuhn, P. H., Lichtenthaler, S. F., Motori, E., Hrelia, S., Wurst, W., Trümbach, D., Langer, T., Krappmann, D., Dittmar, G., Tatzelt, J., & Winklhofer, K. F. (2013). The E3 ligase parkin maintains mitochondrial integrity by increasing linear ubiquitination of NEMO. Molecular cell, 49(5), 908–921. <https://doi.org/10.1016/j.molcel.2013.01.036>
92. Borsche, M., König, I. R., Delcambre, S., Petrucci, S., Balck, A., Brüggemann, N., Zimprich, A., Wasner, K., Pereira, S. L., Avenali, M., Deuschle, C., Badanjak, K., Ghelfi, J., Gasser, T., Kasten, M., Rosenstiel, P., Lohmann, K., Brockmann, K., Valente, E. M., Youle, R. J., … Klein, C. (2020). Mitochondrial damage-associated inflammation highlights biomarkers in PRKN/PINK1 parkinsonism. Brain : a journal of neurology, 143(10), 3041–3051. https://doi.org/10.1093/brain/awaa246
93. Torres-Odio, S., Key, J., Hoepken, H. H., Canet-Pons, J., Valek, L., Roller, B., Walter, M., Morales-Gordo, B., Meierhofer, D., Harter, P. N., Mittelbronn, M., Tegeder, I., Gispert, S., & Auburger, G. (2017). Progression of pathology in PINK1-deficient mouse brain from splicing via ubiquitination, ER stress, and mitophagy changes to neuroinflammation. Journal of neuroinflammation, 14(1), 154. <https://doi.org/10.1186/s12974-017-0928-0>
94. Poláchová, E., Bach, K., Heuten, E., Stanchev, S., Tichá, A., Lampe, P., Majer, P., Langer, T., Lemberg, M. K., & Stříšovský, K. (2023). Chemical Blockage of the Mitochondrial Rhomboid Protease PARL by Novel Ketoamide Inhibitors Reveals Its Role in PINK1/Parkin-Dependent Mitophagy. Journal of medicinal chemistry, 66(1), 251–265. <https://doi.org/10.1021/acs.jmedchem.2c01092>
95. Roverato, N. D., Sailer, C., Catone, N., Aichem, A., Stengel, F., & Groettrup, M. (2021). Parkin is an E3 ligase for the ubiquitin-like modifier FAT10, which inhibits Parkin activation and mitophagy. Cell reports, 34(11), 108857. https://doi.org/10.1016/j.celrep.2021.108857
96. Sarraf SA, Raman M, Guarani-Pereira V, et al. (2013) Landscape of the PARKIN-dependent ubiquitylome in response to mitochondrial depolarization. Nature 496(7445):372–376. <https://doi.org/10.1038/nature12043>
97. Hasson SA, Kane LA, Yamano K, et al. (2013) High-content genome-wide RNAi screens identify regulators of parkin upstream of mitophagy. Nature 504(7479):291–295. <https://doi.org/10.1038/nature12748>
98. Wauer T, Simicek M, Schubert A, et al. (2015) Mechanism of phospho-ubiquitin-induced PARKIN activation. Nature 524(7565):370–374. <https://doi.org/10.1038/nature14879>
99. Schubert AF, Gladkova C, Pardon E, et al. (2017) Structure of PINK1 in complex with its substrate ubiquitin. Nature 552(7683):51–56. <https://doi.org/10.1038/nature24645>
100. Gladkova C, Maslen SL, Skehel JM, et al. (2018) Mechanism of parkin activation by PINK1. Nature 559(7714):410–414. <https://doi.org/10.1038/s41586-018-0224-x>
101. Matheoud D, Cannon T, Voisin A, et al. (2019) Intestinal infection triggers Parkinson’s disease-like symptoms in Pink1-/- mice. Nature 571(7766):565–569. <https://doi.org/10.1038/s41586-019-1405-y>
102. Gan ZY, Callegari S, Cobbold SA, Cotton TR, Mlodzianoski MJ, Schubert AF, Geoghegan ND, Rogers KL, Leis A, Dewson G, Glukhova A, Komander D (2022) Activation mechanism of PINK1. Nature 602(7896):328–335. <https://doi.org/10.1038/s41586-021-04340-2>
103. Shiba-Fukushima K, Ishikawa KI, Inoshita T, Izawa N, Takanashi M, Sato S, Onodera O, Akamatsu W, Okano H, Imai Y, Hattori N (2017) Evidence that phosphorylated ubiquitin signaling is involved in the etiology of Parkinson’s disease. Hum Mol Genet 26(16):3172–3185. <https://doi.org/10.1093/hmg/ddx201>
104. Amo T, Sato S, Saiki S, Wolf AM, Toyomizu M, Gautier CA, Shen J, Ohta S, Hattori N (2011) Mitochondrial membrane potential decrease caused by loss of PINK1 is not due to proton leak, but to respiratory chain defects. Neurobiol Dis 41(1):111–118. https://doi.org/10.1016/j.nbd.2010.08.027
105. Noda S, Sato S, Fukuda T, Tada N, Uchiyama Y, Tanaka K, Hattori N (2020) Loss of Parkin contributes to mitochondrial turnover and dopaminergic neuronal loss in aged mice. Neurobiol Dis 136:104717. <https://doi.org/10.1016/j.nbd.2019.104717>
106. Yamano, K., Wang, C., Sarraf, S. A., Münch, C., Kikuchi, R., Noda, N. N., Hizukuri, Y., Kanemaki, M. T., Harper, W., Tanaka, K., Matsuda, N., & Youle, R. J. (2018). Endosomal Rab cycles regulate Parkin-mediated mitophagy. eLife , 7, e31326. <https://doi.org/10.7554/eLife.31326>
107. Scarffe, L. A., Stevens, D. A., Dawson, V. L., & Dawson, T. M. (2014). Parkin and PINK1: much more than mitophagy. Trends in Neurosciences , 37(6), 315–324. <https://doi.org/10.1016/j.tins.2014.03.004>
108. Liang, H., He, S., Yang, J., Jia, X., Wang, P., Chen, X., Zhang, Z., Zou, X., McNutt, M. A., Shen, W. H., & Yin, Y. (2014). PTENα, a PTEN isoform translated through alternative initiation, regulates mitochondrial function and energy metabolism. Cell Metabolism , 19(5), 836–848. <https://doi.org/10.1016/j.cmet.2014.03.023>
109. Liu, L., Feng, D., Chen, G., Chen, M., Zheng, Q., Song, P., Ma, Q., Zhu, C., Wang, R., Qi, W., Huang, L., Xue, P., Li, B., Wang, X., Jin, H., Wang, J., Yang, F., Liu, P., Zhu, Y., Sui, S., et al. (2012). Mitochondrial outer-membrane protein FUNDC1 mediates hypoxia-induced mitophagy in mammalian cells. Nature Cell Biology , 14(2), 177–185. <https://doi.org/10.1038/ncb2422>
110. Chen, Z., Liu, L., Cheng, Q., Li, Y., Wu, H., Zhang, W., Wang, Y., Sehgal, S. A., Siraj, S., Wang, X., Wang, J., Zhu, Y., & Chen, Q. (2017). Mitochondrial E3 ligase MARCH5 regulates FUNDC1 to fine-tune hypoxic mitophagy. EMBO Reports , 18(3), 495–509. <https://doi.org/10.15252/embr.201643309>
111. Gao F, Chen D, Si J, Hu Q, Qin Z, Fang M, Wang G (2015) The mitochondrial protein BNIP3L is the substrate of PARK2 and mediates mitophagy in PINK1/PARK2 pathway. Human Molecular Genetics 24(9):2528–2538. <https://doi.org/10.1093/hmg/ddv017>
112. Soutar MPM, Melandri D, O'Callaghan B, Annuario E, Monaghan AE, Welsh NJ, D'Sa K, Guelfi S, Zhang D, Pittman A, Trabzuni D, Verboven AHA, Pan KS, Kia DA, Bictash M, Gandhi S, Houlden H, Cookson MR, Kasri NN, Wood NW, Plun-Favreau H (2022) Regulation of mitophagy by the NSL complex underlies genetic risk for Parkinson's disease at 16q11.2 and MAPT H1 loci. Brain: A Journal of Neurology 145(12):4349–4367. <https://doi.org/10.1093/brain/awac325>
113. Moskal N, Riccio V, Bashkurov M, Taddese R, Datti A, Lewis PN, Angus McQuibban G (2020) ROCK inhibitors upregulate the neuroprotective Parkin-mediated mitophagy pathway. Nature Communications 11(1):88. <https://doi.org/10.1038/s41467-019-13781-3>
114. Shiba-Fukushima K, Inoshita T, Sano O, Iwata H, Ishikawa KI, Okano H, Akamatsu W, Imai Y, Hattori N (2020) A Cell-Based High-Throughput Screening Identified Two Compounds that Enhance PINK1-Parkin Signaling. iScience 23(5):101048. <https://doi.org/10.1016/j.isci.2020.101048>
115. Wang Y, Luo S, Su H, Wang Z, Chu L, Zhang C (2024) BL-918 activates PINK1/Parkin signaling pathway to ameliorate the progression of Parkinson's disease. The Journal of Biological Chemistry 300(8):107543. <https://doi.org/10.1016/j.jbc.2024.107543>
116. Rusilowicz-Jones E V, Jardine J, Kallinos A, Pinto-Fernandez A, Guenther F, Giurrandino M, Barone F G, McCarron K, Burke C J, Murad A, Martinez A, Marcassa E, Gersch M, Buckmelter A J, Kayser-Bricker K J, Lamoliatte F, Gajbhiye A, Davis S, Scott H C, Murphy E, Clague M J (2020) USP30 sets a trigger threshold for PINK1-PARKIN amplification of mitochondrial ubiquitylation. Life Science Alliance 3(8):e202000768. <https://doi.org/10.26508/lsa.202000768>
117. Bingol B, Tea JS, Phu L, Reichelt M, Bakalarski CE, Song Q, Foreman O, Kirkpatrick DS, Sheng M (2014) The mitochondrial deubiquitinase USP30 opposes parkin-mediated mitophagy. Nature 510(7505):370–375. <https://doi.org/10.1038/nature13418>
118. Okarmus J, Agergaard JB, Stummann TC, Haukedal H, Ambjørn M, Freude KK, Fog K, Meyer M (2024) USP30 inhibition induces mitophagy and reduces oxidative stress in parkin-deficient human neurons. Cell Death & Disease 15(1):52. <https://doi.org/10.1038/s41419-024-06439-6>
119. Larson-Casey JL, He C, Carter AB (2020) Mitochondrial quality control in pulmonary fibrosis. Redox Biology 33:101426. <https://doi.org/10.1016/j.redox.2020.101426>
120. Zerihun M, Sukumaran S, Qvit N (2023) The Drp1-Mediated Mitochondrial Fission Protein Interactome as an Emerging Core Player in Mitochondrial Dynamics and Cardiovascular Disease Therapy. International Journal of Molecular Sciences 24(6):5785. <https://doi.org/10.3390/ijms24065785>
121. Haeussler S, Köhler F, Witting M, Premm MF, Rolland SG, Fischer C, Chauve L, Casanueva O, Conradt B. Autophagy compensates for defects in mitochondrial dynamics. PLoS Genet 16(3): e1008638. <https://doi.org/10.1371/journal.pgen.1008638>
122. Zhao Q, Niu Q, Chen J, Xia T, Zhou G, Li P, Dong L, Xu C, Tian Z, Luo C, Liu L, Zhang S, Wang A. Roles of mitochondrial fission inhibition in developmental fluoride neurotoxicity: mechanisms of action in vitro and associations with cognition in rats and children. Arch Toxicol 93(3): 709–726. <https://doi.org/10.1007/s00204-019-02390-0>
123. Xian H, Liou YC. Functions of outer mitochondrial membrane proteins: mediating the crosstalk between mitochondrial dynamics and mitophagy. Cell Death Differ 28(3): 827–842. <https://doi.org/10.1038/s41418-020-00657-z>
124. Twig G, Shirihai OS. The interplay between mitochondrial dynamics and mitophagy. Antioxid Redox Signal 14(10): 1939–1951. <https://doi.org/10.1089/ars.2010.3779>
125. Xiong W, Ma Z, An D, Liu Z, Cai W, Bai Y, Zhan Q, Lai W, Zeng Q, Ren H, Xu D. Mitofusin 2 Participates in Mitophagy and Mitochondrial Fusion Against Angiotensin II-Induced Cardiomyocyte Injury. Front Physiol 10: 411. <https://doi.org/10.3389/fphys.2019.00411>
126. Peng K, Xiao J, Yang L, Ye F, Cao J, Sai Y. Mutual Antagonism of PINK1/Parkin and PGC-1α Contributes to Maintenance of Mitochondrial Homeostasis in Rotenone-Induced Neurotoxicity. Neurotoxicity Res 35(2): 331–343. <https://doi.org/10.1007/s12640-018-9957-4>
127. Kumar M, Acevedo-Cintrón J, Jhaldiyal A, Wang H, Andrabi SA, Eacker S, Karuppagounder SS, Brahmachari S, Chen R, Kim H, Ko HS, Dawson V L, Dawson T M. Defects in Mitochondrial Biogenesis Drive Mitochondrial Alterations in PARKIN-Deficient Human Dopamine Neurons. Stem Cell Reports 15(3): 629–645. <https://doi.org/10.1016/j.stemcr.2020.07.013>
128. Lurette O, Martín-Jiménez R, Khan M, Sheta R, Jean S, Schofield M, Teixeira M, Rodriguez-Aller R, Perron I, Oueslati A, Hebert-Chatelain E. Aggregation of alpha-synuclein disrupts mitochondrial metabolism and induce mitophagy via cardiolipin externalization. Cell Death Dis 14(11): 729. <https://doi.org/10.1038/s41419-023-06251-8>
129. Hui S, George J, Kapadia M, Chau H, Bariring Z, Earnshaw R, Shafiq K, Kalia LV, Kalia SK. Mitophagy Upregulation Occurs Early in the Neurodegenerative Process Mediated by α-Synuclein. Mol Neurobiol 61(11): 9032–9042. <https://doi.org/10.1007/s12035-024-04131-6>
130. Kinnart I, Manders L, Heyninck T, Imberechts D, Praschberger R, Schoovaerts N, Verfaillie C, Verstreken P, Vandenberghe W. Elevated α-synuclein levels inhibit mitophagic flux. NPJ Parkinson's Dis. 2024;10(1):80. <https://doi.org/10.1038/s41531-024-00696-0>
131. Martinez JH, Alaimo A, Gorojod RM, Porte Alcon S, Fuentes F, Coluccio Leskow F, Kotler ML. Drp-1 dependent mitochondrial fragmentation and protective autophagy in dopaminergic SH-SY5Y cells overexpressing alpha-synuclein. Molecular and Cellular Neurosciences. 2018;88:107–117. <https://doi.org/10.1016/j.mcn.2018.01.004>
132. Shaltouki A, Hsieh CH, Kim MJ, Wang X. Alpha-synuclein delays mitophagy and targeting Miro rescues neuron loss in Parkinson's models. Acta Neuropathologica. 2018;136(4):607–620. <https://doi.org/10.1007/s00401-018-1873-4>
133. Chen J, Ren Y, Gui C, Zhao M, Wu X, Mao K, Li W, Zou F. Phosphorylation of Parkin at serine 131 by p38 MAPK promotes mitochondrial dysfunction and neuronal death in mutant A53T α-synuclein model of Parkinson's disease. Cell Death & Disease. 2018;9(6):700. <https://doi.org/10.1038/s41419-018-0722-7>
134. Yin S, Shen M, Zhang Y, Wu J, Song R, Lai X, Tian Z, Wang T, Jin W, Yan J. Nur77 increases mitophagy and decreases aggregation of α-synuclein by modulating the p-c-Abl/p-PHB2 Y121 in α-synuclein PFF SH-SY5Y cells and mice. European Journal of Medicinal Chemistry. 2024;268:116251. <https://doi.org/10.1016/j.ejmech.2024.116251>
135. Wang W, Han R, He HJ, Li J, Chen SY, Gu Y, Xie C. Administration of quercetin improves mitochondria quality control and protects the neurons in 6-OHDA-lesioned Parkinson's disease models. Aging. 2021 Apr 20;13(8):11738–11751. <https://doi.org/10.18632/aging.202868>
136. Lu Y, Gao L, Yang Y, Shi D, Zhang Z, Wang X, Huang Y, Wu J, Meng J, Li H, Yan D. Protective role of mitophagy on microglia-mediated neuroinflammatory injury through mtDNA-STING signaling in manganese-induced parkinsonism. Journal of Neuroinflammation. 2025;22(1):55. <https://doi.org/10.1186/s12974-025-03396-5>
137. Quinn PMJ, Moreira PI, Ambrósio AF, Alves CH. PINK1/PARKIN signalling in neurodegeneration and neuroinflammation. Acta Neuropathologica Communications. 2020;8(1):189. <https://doi.org/10.1186/s40478-020-01062-w>
138. Qiu J, Chen Y, Zhuo J, Zhang L, Liu J, Wang B, Sun D, Yu S, Lou H. Urolithin A promotes mitophagy and suppresses NLRP3 inflammasome activation in lipopolysaccharide-induced BV2 microglial cells and MPTP-induced Parkinson's disease model. Neuropharmacology. 2022;207:108963. <https://doi.org/10.1016/j.neuropharm.2022.108963>
139. Hu ZL, Sun T, Lu M, Ding JH, Du RH, Hu G. Kir6.1/K-ATP channel on astrocytes protects against dopaminergic neurodegeneration in the MPTP mouse model of Parkinson's disease via promoting mitophagy. Brain, Behavior, and Immunity. 2019;81:509–522. <https://doi.org/10.1016/j.bbi.2019.07.009>
140. Motawi TK, Al-Kady RH, Senousy MA, Abdelraouf SM (2023) Repaglinide Elicits a Neuroprotective Effect in Rotenone-Induced Parkinson's Disease in Rats: Emphasis on Targeting the DREAM-ER Stress BiP/ATF6/CHOP Trajectory and Activation of Mitophagy. ACS Chemical Neuroscience 14(1):180–194. <https://doi.org/10.1021/acschemneuro.2c00656>
141. Li X, Li W, Xie X, Fang T, Yang J, Shen Y, Wang Y, Wang H, Tao L, Zhang H (2025) ROS Regulate Rotenone-induced SH-SY5Y Dopamine Neuron Death Through Ferroptosis-mediated Autophagy and Apoptosis. Molecular Neurobiology. Advance online publication. <https://doi.org/10.1007/s12035-025-04824-6>
142. Zhang Y, Zhang B (2024) Bifenthrin Caused Parkinson's-Like Symptoms Via Mitochondrial Autophagy and Ferroptosis Pathway Stereoselectively in Parkin−/− Mice and C57BL/6 Mice. Molecular Neurobiology 61(11):9694–9707. <https://doi.org/10.1007/s12035-024-04140-5>
143. Tian Y, Lu J, Hao X, Li H, Zhang G, Liu X, Li X, Zhao C, Kuang W, Chen D, Zhu M (2020) FTH1 Inhibits Ferroptosis Through Ferritinophagy in the 6-OHDA Model of Parkinson's Disease. Neurotherapeutics 17(4):1796–1812. <https://doi.org/10.1007/s13311-020-00929-z>
144. Yokochi M. (2000). Development of the nosological analysis of juvenile parkinsonism. Brain & development, 22 Suppl 1, S81–S86. <https://doi.org/10.1016/s0387-7604(00)00131-5>
145. Antico, O., Thompson, P. W., Hertz, N. T., Muqit, M. M. K., & Parton, L. E. (2025). Targeting mitophagy in neurodegenerative diseases. Nature reviews. Drug discovery, 24(4), 276–299. <https://doi.org/10.1038/s41573-024-01105-0>
146. Zhiguo, F., Ji, W., Shenyuan, C., Guoyou, Z., Chen, K., Hui, Q., Wenrong, X., & Zhai, X. (2023). A swift expanding trend of extracellular vesicles in spinal cord injury research: a bibliometric analysis. Journal of nanobiotechnology, 21(1), 289. https://doi.org/10.1186/s12951-023-02051-6
